# Multitask computation through dynamics in recurrent spiking neural networks

**DOI:** 10.1038/s41598-023-31110-z

**Published:** 2023-03-10

**Authors:** Mechislav M. Pugavko, Oleg V. Maslennikov, Vladimir I. Nekorkin

**Affiliations:** Institute of Applied Physics of the Russian Academy of Sciences, Nizhny Novgorod, 603950 Russia

**Keywords:** Complex networks, Nonlinear phenomena

## Abstract

In this work, inspired by cognitive neuroscience experiments, we propose recurrent spiking neural networks trained to perform multiple target tasks. These models are designed by considering neurocognitive activity as computational processes through dynamics. Trained by input–output examples, these spiking neural networks are reverse engineered to find the dynamic mechanisms that are fundamental to their performance. We show that considering multitasking and spiking within one system provides insightful ideas on the principles of neural computation.

## Introduction

Viewing the brain as a complex neuronal network (the connectome) and its emerging cognitive activity as a multidimensional and multilevel dynamic process (the cognitome) has gradually become an increasingly important physico-mathematical description of this biocognitive enigma^1–5^. There have been many data-driven and theory-motivated approaches that aim to bridge experimentally found neural connections and measured spike trains with observed phenomena at cognitive and behavioral levels^6–10^. Various models in the form of large-scale networks of coupled dynamic neuron-like units have been used to understand fundamental principles. Different biological constraints and detail levels have been applied to the design of such models^11–14^. An important distinction between these models is whether a system is obtained via a bottom-up or top-down approach^15^. In the former case, one starts from some microscopic connectivity structure and biophysical or simplified neuron models and seeks to find features leading to some population patterns of interest. An undoubted advantage of this approach is its neurophysiological similarity to the biological prototype; however, even the most complicated models of this class lack insight into the emergence of cognitive-like behaviors. Models in the latter case take an opposite approach; i.e., the cognitive task or function of interest is explicitly formulated, often in a reduced form, and then an artificial neural network with possible biological constraints is machine-trained to produce the target function^16–18^. Although the model system, usually in the form of a recurrent neural network, may lack microscopic similarity with brain networks, it can serve as a practical tool for relating neural dynamics and cognitive processes. The resulting neural network is a multidimensional system that can be studied by the methods of nonlinear dynamics and complex network theory, and the structure and dynamic mechanisms responsible for the observed functions can be revealed^19–23^. This approach has been shown to provide insights into the neural population-level explanations of a number of cognitive functions, including decision-making and working memory.

The vast majority of studies examine neural networks trained to perform one particular task, and the models they use are based on the rate description where the neurons are presented as nonlinear activation functions of the weighted sum of inputs. A prominent feature of biological neural networks is the possibility of performing multiple tasks, so uncovering how the same artificial neural network completes different tasks can help to understand these undisclosed mechanisms in the biological prototype^24–26^. The spiking dynamics produced by communicating neurons is another biological property neglected by most models of this type^27–32^. Moreover, spiking networks have been actively studied in recent years in machine learning and neuromorphic engineering communities due to their promising energy capabilities for next-generation artificial neural networks^33,34^. In this work, we propose spiking recurrent neural networks that are trained to perform several target tasks inspired by experimental settings in cognitive neuroscience^16,35–40^. An important point is that the trained network is considered a multidimensional dynamical system that is reverse-engineered by nonlinear dynamic methods to uncover the principles underlying the performed tasks. These principles are formulated both in terms of population trajectories in the activity space of neural networks and in terms of specialized and mixed-selective clusters responsible for completing different tasks.

## Methods

To construct a functional spiking network capable of implementing multiple tasks inspired by cognitive neuroscience, we elaborate our model in the framework of recurrent neural networks trained by machine learning methods to produce context-dependent target functions. Considering the resulting neural network as a dynamical system, we study its structural and dynamic features supporting the observed functions. In this section, we describe the basic issues of the network design and the methods for revealing the dynamic mechanisms that are fundamental to its performance in cognitive-inspired tasks. In “Model” section, we consider the network architecture and the model neuron used to build the spiking neural network in our work. “Cognitive tasks” section describes the target tasks motivated by cognitive neuroscience experiments that are used for training the spiking neural network. In “Training procedure” section, the issues relating to the learning method, the loss function calculation and the training procedure are explained. Finally, “Analysis of the trained network” section is devoted to the basic methods for evaluating the network accuracy, analyzing its population dynamics and determining the role of individual neurons in performing the target tasks.

### Model

#### Network architecture and target functions

**Figure 1:**
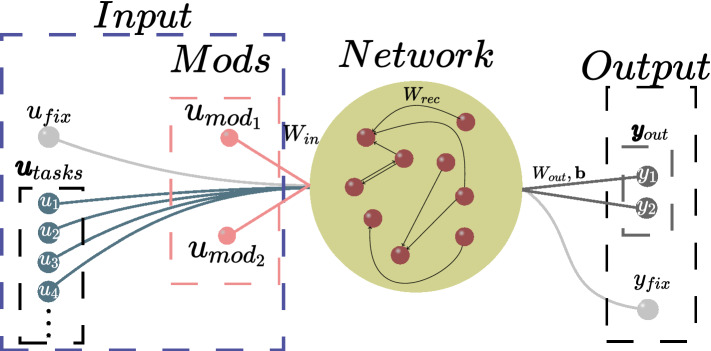
Architecture of the spiking neural network. The input layer sends three distinct classes of inputs—the fixation signal $u_{fix}$, the sensory inputs of two modalities ${\textbf{u}}_{mod}=({u}_{mod_1},{u}_{mod_2})$, and the task coding one-hot vector ${\textbf{u}}_{tasks}$—through the input matrix ${\textbf{W}}^{in}$. The central element—the spiking recurrent neural network—is characterized by the connectivity matrix ${\textbf{W}}^{rec}$. Its activity is output via matrix ${\textbf{W}}^{out}$ and vector ${\textbf{b}}$ by the output response units ${\textbf{y}}_{out}=(y_1,y_2)$ and fixation $y_{fix}$.

The network architecture we use to construct our model is shown in Fig. 1. It consists of the input layer, the central recurrent neural network, and the output layer. The recurrent neural network consists of *N* spiking neurons whose structure of connections is given by the weight matrix ${\textbf{W}}^{rec} \in {\mathbb {R}}^{N \times N}$. Initially, the network is all-to-all coupled, and the entries of ${\textbf{W}}^{rec}$ are randomly chosen from the normal distribution $\sqrt{2/N} {\mathscr {N}}(0, 1)$. All the diagonal entries are kept at zero throughout the entire simulation to prevent neural self-excitation.

The input stimuli $u_k(t)$ ($k=1,\ldots ,N_{in}$) arrive at the recurrent neural network through the input matrix ${\textbf{W}}^{in} \in {\mathbb {R}}^{N \times N_{in}}$, where $N_{in}$ is the number of inputs. The entries of this matrix are initialized in a way that is identical to ${\textbf{W}}^{rec}$. The output responses $y_j(t)$ ($j=1,\ldots ,N_{out}$) are produced by the network activity via the matrix ${\textbf{W}}^{out} \in {\mathbb {R}}^{N_{out} \times N}$ and the bias vector ${\textbf{b}} \in {\mathbb {R}}^{N_{out}}$, where $N_{out}$ is the number of outputs. The entries of ${\textbf{W}}^{out}$ and ${\textbf{b}}$ are initialized according to the uniform distribution ${\mathscr {U}}(-\sqrt{1 / N}, \sqrt{1 / N})$.

**Figure 2:**
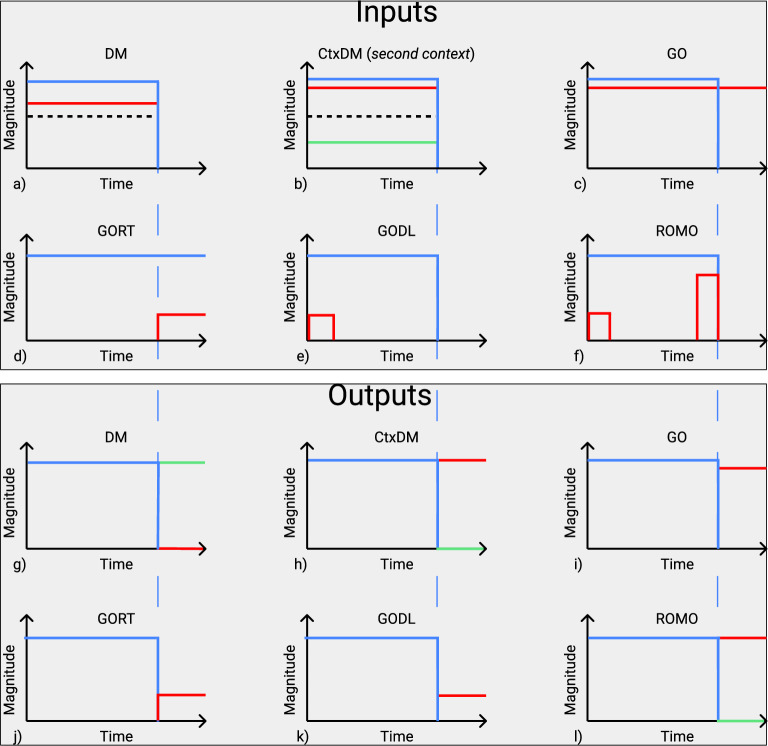
Target tasks used to train the spiking neural network. The top subplots (**a**–**f**) show the inputs, where the blue lines correspond to the fixation input $u_{fix}$ and the red and green lines show the input stimuli $u_{mod_1}$ and $u_{mod_2}$, respectively. The bottom subplots (**g**–**l**) show the target outputs, where the blue lines indicate the fixation output $y_{fix}$ and the red and green lines show outputs $y_1$ and $y_2$, respectively. (**a**) Decision-making (*DM*) task: if the input stimulus $u_1$ is higher on average than the threshold (the dotted line), then the target outputs are $y_1=0$ and $y_2=1$ (**g**). Otherwise, $(y_1, y_2) = (1, 0)$. (**b**) Context decision-making (*CtxDM*) task: the context signal indicates which of the stimuli $u_1$ or $u_2$ should be compared with the threshold (the dotted line). The outputs (**h**) are analogous to *DM*. (**c**) Inputs and (**i**) corresponding outputs of the go task (*Go*). (**d**) Inputs and (**j**) corresponding outputs of the go task with reaction times (*GoRt*). (**e**) Inputs and (**k**) corresponding outputs of the delayed go task (*GoDl*). (**f**) Working memory Romo task (*Romo*): if the second input stimulus is larger than the first one, then the target output (**l**) is $(y_1, y_2) = (1, 0)$; otherwise, $(y_1, y_2) = (0, 1)$. The blue vertical lines show the termination of the fixation phase.

The target functions used to train our network are inspired by the cognitive tasks described below. Each of them is based on a certain mapping of inputs $u_k(t)$ to outputs $y_j(t)$ during the trial; see Fig. 2. The input signals $u_k(t)$ input into the network can be divided into three distinct classes. The first class, given by a binary value (0 or 1), is the so-called fixation signal $u_{fix}(t) \in {\mathbb {R}}^1$, which marks out two phases in each trial—the stimulus phase of the input perception (where $u_{fix}=1$) and the response phase of the motor output (where $u_{fix}=0$). The second class contains two noisy scalar values $(u_{mod_1}, u_{mod_2})$ (or one vector ${\textbf{u}}_{mod}$), which provide sensory inputs of two modalities to the network. Each of these sensory signals can be presented as the sum of the deterministic part $u_0$ and the additive white Gaussian noise of standard deviation $\sigma$: $u_{mod}=u_0 + {\mathscr {N}}(0, \sigma )$. The third class is the task coding input presented by the one-hot vector ${\textbf{u}}_{tasks} = (u_{task_1}, u_{task_2},..., u_{task_{N_{tasks}}})^T$, where $N_{tasks}$ is the number of target tasks. During each trial, the unit of ${\textbf{u}}_{tasks}$ corresponding to the current task is equal to 1, while the other units are 0. Thus, all the inputs of the spiking neural network can be described by the following vector

1 $$\begin{aligned} {\textbf{u}} = (u_{fix}, {\textbf{u}}_{mod}, {\textbf{u}}_{tasks})^T \end{aligned}$$

whose dimension equals $N_{in} = N_{tasks} + 3$.

The outputs are presented by two response signals $(y_{1}, y_{2})={\textbf{y}}_{out}$ and one fixation signal $y_{fix}$, which produce activity by reading out the recurrent network dynamics. The target of the fixation output is to simply replicate the fixation input. The way the network should map the stochastic inputs $u_{mod_1}(t), u_{mod_2}(t)$ to the output signals $y_{1}(t), y_{2}(t)$ varies for different tasks and is defined in each trial by the task coding vector.

Input–output transformations for different target tasks are schematically shown in Fig. 2 for noiseless inputs. The upper part of the plot contains input signals for different tasks denoted by abbreviations, and the corresponding target outputs are given in the bottom part. The blue lines denote the fixation inputs and outputs in the upper and bottom parts, respectively; the red and green lines define the sensory inputs and motor outputs. It should be noted that the inputs $u_{mod_1}(t), u_{mod_2}(t)$ used during training and testing have additive noise terms, so the deterministic plots in Fig. 2a–f are given only for illustration purposes.

A detailed description of the prototype neuroscience experiments is given below, and here, we briefly describe the input–output transformations used as target functions during training.

- DM (Decision making task)
  During the stimulus phase, one input $u_{mod_1}(t)$ (or $u_{mod_2}(t)$) is selected from a noisy signal with the mean chosen between 0 and 1, and the target output is $(y_1, y_2) = (0, 0)$. During the response phase, the inputs are zero, the target outputs are $(y_1, y_2) = (1, 0)$ if the time average of $u_{mod_1}(t)$ (or $u_{mod_2}(t)$) is below the threshold $u_{th} = 0.5$, and $(y_1, y_2) = (0, 1)$ otherwise; see Fig. 2a,g.
- CtxDM (Context decision-making task)
  During the stimulus phase, $u_{mod_1}(t)$ and $u_{mod_2}(t)$ are selected from two noisy signals with particular means and the context input ${\textbf{u}}_{tasks}$ reports, and one of the two values should be compared with the threshold $u_{th}$ while the other should be ignored. During the response phase, the inputs are zero, and the target output is $(y_1, y_2) = (1, 0)$ if the time average of the input of interest is below the threshold $u_{th} = 0.5$ and $(y_1, y_2) = (0, 1)$; otherwise, see Fig. 2b,h.
- Romo (Working memory Romo task)
  During the stimulus phase, one input $u_{mod_1}(t)$ (or $u_{mod_2}(t)$) is selected from two consecutive rectangular pulses separated by some time delay. In the response phase, the target output is $(y_1, y_2) = (1, 0)$ when the first stimulus is larger than the second and $(y_1, y_2) = (0, 1)$ otherwise; see Fig. 2f,l.
- Go (Go/no go task)
  During the whole trial, one input $u_{mod_1}(t)$ (or $u_{mod_2}(t)$) is selected from a noisy signal with a certain mean between 0 and 1. In the response phase, the corresponding output $y_1$ (or $y_2$) is expected to generate a constant signal whose magnitude is equal to the input mean; see Fig. 2c,i
- GoRt (Reaction time go task)
  During the whole trial, the fixation input equals one, and initially, all the inputs are zero. At some random moment of the trial, one noisy input $u_{mod_1}(t)$ (or $u_{mod_2}(t)$) is switched on with some mean, and the target is to generate the same-magnitude signal at the output $y_1$ (or $y_2$) of the corresponding modality; see Fig. 2d,j.
- GoDl (Delayed version of the go task)
  During the stimulus phase, one input $u_{mod_1}(t)$ (or $u_{mod_2}(t)$) produces a short rectangular pulse of magnitude between 0 and 1. In the response phase, the corresponding output $y_1$ (or $y_2$) is expected to generate the same-magnitude signal when the fixation input vanishes; see Fig. 2e,k.

#### Neuron model

As a spiking neuron model, we consider the adaptive exponential (AdEx) integrate-and-fire neuron given by the following system^41^:

2 $$\begin{aligned} {\left\{ \begin{array}{ll} \frac{dv}{dt} &{} = {\frac{1}{\tau _m}} \left(-v + I + \theta \exp {\left(\frac{v - v_{th}}{\theta }\right)} - a\right), \\ \frac{da}{dt} &{} = {\frac{1}{\tau _a}}(a_{current} v - a), \\ \frac{dI}{dt} &{} = { - \frac{1}{\tau _s}(I+I^{out})}, \end{array}\right. } \end{aligned}$$

where *v* is a membrane potential, *a* is an adaptation variable, and *I* is a synaptic current. Parameter $\tau _m$ is a time constant that characterizes the relaxation rate of the membrane potential, $\tau _a$ is the adaptation time, $\tau _s$ is the synaptic time, $a_{current}$ is the adaptation coupling parameter, and $\theta$ is the sharpness of the exponential nonlinearity. When the membrane potential exceeds the threshold value $v_{th}$, it resets to $v\rightarrow v_{reset}$, and the adaptation variable resets to $a \rightarrow a + a_s$, where $v_{reset}$ and $a_s$ are the parameters. The synaptic current *I*(*t*) takes into account stimulation $I^{out}=I^{in}+I^{rec}$ from input connections $I^{in}$ and recurrent links $I^{rec}$, and in their absence, it relaxes to zero.

**Figure 3:**
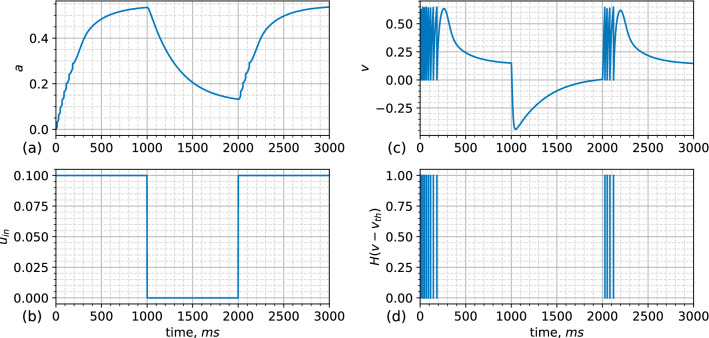
Adaptive exponential neuron (2) under rectangular input pulses: dynamics of (**a**) the adaptation variable *a*(*t*), (**b**) the injected input current $u_{in}$, (**c**) the membrane potential *v*(*t*), and (**d**) the resulting spike sequence determined by the Heaviside step function *H*(*x*). The control parameters are given in Table 1.

An example of neuron dynamics under the input current is shown in Fig. 3. The adaptation variable is gradually increased (Fig. 3a) under the influence of the injected rectangular pulse current (Fig. 3b); moreover, the membrane potential produces fast oscillations (Fig. 3c), giving rise to spikes (Fig. 3d). During the duration of the stimulus, the variable *a* increases while the spike firing rate decreases until spike generation is completely stopped. After switching off the input current, the adaptation variable decreases, leading to the reappearance of the neuron’s ability to generate spikes. The neuron model parameters taken for drawing this plot, which are mostly used in the current work, are presented in Table 1.

**Table 1:** Parameters of the AdEx neuron model used in the current research.

Label	Value
$\tau _a$	2 s
$\tau _m$	10 ms
$\tau _s$	5 ms
$v_{th}$	0.65 mV ($\forall N \ne 256$), 0.45 mV ($N=256$)
$v_{reset}$	0 mV
$\theta$	0.5 mV
$a_s$	0.02 nA
$a_{current}$	4 ns

#### Spiking neural network

During computer simulations, the network of adaptive exponential neurons (2) is described in a discrete form obtained by the Euler method as follows:

3 $$\begin{aligned} {\left\{ \begin{array}{ll} v_j(n + 1) &{} = v_j(n) + \frac{\Delta t}{\tau _m}\left\{ -v_j(n) + \theta \exp {\left[ \frac{v_j(n)- v_{th}}{\theta }\right] } + I_j(n) - a_j(n)\right. \}, \\ a_j(n + 1) &{} = a_j(n) + \frac{\Delta t}{\tau _a}\left\{ a_j^{current}v_j(n) - a_j(n)\right\} , \\ I_j(n + 1) &{} = I_j(n) \left( 1 - \frac{\Delta t}{\tau _s}\right) + I_j^{in}(n + 1) + I_j^{rec}(n + 1), \\ I_j^{in}(n + 1) &{} = \sum \limits _{k=1}^{N_{in}} w^{in}_{jk}u_k(n + 1), \\ I_j^{rec}(n + 1) &{} = \sum \limits _{k=1}^{N} w^{rec}_{jk}z_k(n), \quad j=1,\ldots ,N, \\ \end{array}\right. } \end{aligned}$$

where $n \in {\mathbb {Z}}$ is the discrete time, $\Delta t=1$ ms, $I_j^{in}$ and $I_k^{rec}$ are the input and recurrent currents, respectively, for neuron $j=1,\ldots ,N$, and $w_{jk}^{in}$ and $w_{jk}^{rec}$ are the entries of weight matrices ${\textbf{W}}^{in}$ and ${\textbf{W}}^{rec}$, respectively. The terms $z_k = H(v_k-v_{th})$ are the spike detecting variables, where *H*(*x*) is the Heaviside step function; that is, $H(x) = 0$ if $x < 0$, and $H(x) = 1$ otherwise. The network outputs are obtained by the exponential filters, which smooth the effect of the spike trains:

4 $$\begin{aligned} y_j(n + 1) = \kappa y_j(n) + \sum _{k = 1}^N w^{out}_{jk}z_k(n) + b_j, \quad j=1,\ldots ,N_{out}, \end{aligned}$$

where $\kappa = \exp {(-\Delta t / \tau _{out})}$ is the filter parameter, $\tau _{out}$ is the filtration time, and $w_{jk}^{out}$ and $b_j$ are the entries of the weight matrix ${\textbf{W}}^{out}$ and the bias vector ${\textbf{b}}$, respectively.

### Cognitive tasks

We consider a family of target tasks inspired by a series of cognitive neuroscience experiments that are similar to those in^24^ and study how a single spiking neural network can implement them. The trial of each task consists of two phases, stimulus and response, where the fixation input is equal to 1 during the first phase and equal to 0 during the second phase. We consider 6 types of cognitive tasks, which are implemented through one of the two stimulus inputs $(u_{mod_1}, u_{mod_2})$, giving rise to a total of 12 different target tasks. These are the decision-making (*DM*) task, the decision-making with a context signal (*CtxDM*) task, the working memory Romo (*Romo*) task, the go/no-go (*Go*) task, the go/no-go with delay (*GoDl*) task, and the go/no-go with a reaction time (*GoRt*) task. Thus, the task coding input is given by ${\textbf{u}}$. These tasks can be classified into two classes—choice tasks (*DM*, *CtxDM*, *Romo*) and repeat tasks (*Go*, *GoRt*, *GoDl*). For the former, the network is trained to produce a binary output indicating which of the input stimuli possesses a target feature (e.g., which is the largest or greater than a threshold). For the latter tasks, the network is trained to reproduce an input stimulus with some additional requirements. Below, we describe in detail the basic features of the cognitive neuroscience experiments and the target input–output transformations used in our model framework.

**Figure 4:**
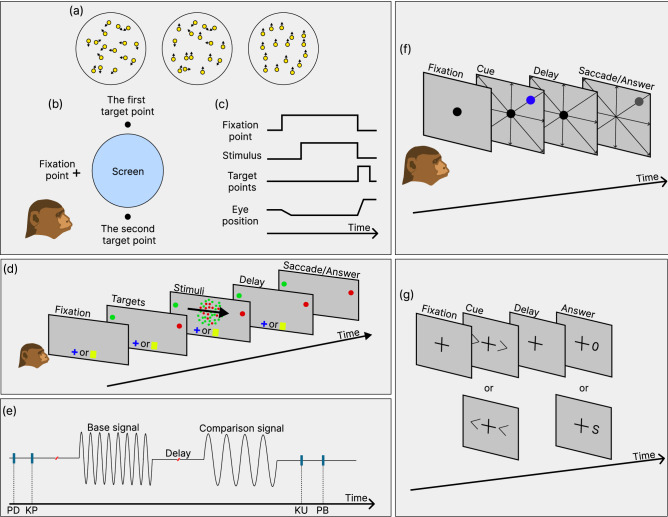
Schematically shown experimental prototypes of the target tasks used to train the spiking neural network: (**a**)–(**c**) the decision-making task, (**d**) the context decision-making task, (**e**) the working memory task, and (**f**,**g**) the go/no-go tasks. (**a**) Random-dot motion visual stimulus with different ratios of coherently moving dots. (**b**) A monkey observing the screen and the positions of the fixation and target points. (**c**) Sequence of events during the trial. The figure is modified from^38^. (**d**) Context decision-making task^16^. First, the context signal turns on (blue cross or yellow square), and then two target (red and green) points appear. After that, a random-dot diagram is shown with a controlled predominant color and the direction (described by an arrow) of coherently moving dots. After the delay period, the animal is expected to respond by using saccadic eye movements to follow the preferential color or direction depending on the context signal. (**e**) Working memory Romo task^37^. First, the probe is placed in the monkey’s hand (PD); then, the monkey places its free hand on a stationary key (KD), after which two vibrotactile stimuli with different frequencies are given separated by a delay. Finally, the monkey releases the key (KU) and presses one of two push buttons to indicate its choice of which stimulus had a higher frequency (PB). (**f**) Example of the go/no-go task^35^. First, the fixation point is highlighted on the screen, and then a cue appears at one of 8 or 4 peripheral locations. After the delay period, the monkey is expected to saccade to the indicated target. (**g**) Example of the go/no-go task^39^. First, human subjects are presented with a fixation visual stimulus, and then one of two possible cues is turned on. After the delay period, the go or no-go signal appears, indicating whether the subject should or should not press the corresponding button in their hand.

**DM** In this traditional two-alternative forced choice task, the laboratory animal is trained to make a motor decision based on the features of the sensory stimulus. For example, in the experiments reported in Ref.^38^, during the trial, a trained monkey is first presented a fixation point on the screen; then, a random-dot motion picture appears, where a controlled fraction of dots move in one of two possible directions while others move randomly; see Fig. 4a–c. After that, the animal is presented two target points and expected to saccade to the direction of the preferred motion. The control parameters are the fraction of coherently moving dots and the trial time. In our reduced setting, during the stimulus phase, the input $u_{mod_1}$ (or $u_{mod_2}$) is presented in the form of a stochastic signal with a mean value between 0 and 1, and the network is trained to determine whether its trial average is below or above the threshold $u_{th} = 0.5$. During the response phase, the target output is $(y_1, y_2) = (1, 0)$ in the former case and $(y_1, y_2) = (0, 1)$ in the latter case; see Fig. 2a,g. The noise intensity and the input proximity to the threshold reflect the level of uncertainty in the experiments.

**CtxDM** The context decision-making task^16^ differs from the previous task in that the random dots are colored either red or green, and depending on the context signal, the laboratory animal should extract one of two possible features—either the preferred direction of dot motion or the predominant color; see Fig. 4d. In our model setting, the two features are presented as two input stimuli combined in ${\textbf{u}}_{mod}$, and the context input ${\textbf{u}}_{tasks}$ reports which one of the two should be compared with the threshold $u_{th}$ while the other should be ignored; see Fig. 2b,h.

**Romo** In the working memory task, which is referred to as the Romo task^36,37^, the trained monkey performs a comparison between two vibrotactile stimuli presented with a time delay; see Fig. 4e. The first—base—and the second—comparison—stimuli applied to the animal’s finger have different frequencies, and the monkey chooses the highest one by pressing one of two buttons. In our model, during the stimulus phase, the network receives two consecutive pulses separated by some time delay via one of the sensory inputs. In the response phase, the network is trained to generate an output $(y_1, y_2) = (1, 0)$ in the case where the first stimulus is larger than the second, and $(y_1, y_2) = (0, 1)$ otherwise; see Fig. 2f,l.

**Go tasks** This is a family of tasks where a cognitive subject responds by a motor action (e.g., a saccadic eye movement or a button press) to the appearance of some predefined stimulus (a go response) while ignoring stimuli with improper features (a no-go response). For example, in the experiment reported in Ref.^35^, the monkey is shown a visual cue on the screen at one of several possible locations, and after a silent period of delay, the animal responds by gazing in the same direction (see Fig. 4f). In the other experiment^39,40^, human subjects were presented with the cue stimulus and selected either a right or left clicker. After a delay time, a go or no-go signal appears on the screen indicating whether the subject in fact should or should not do complete the action (see Fig. 4g). Based on these examples, we consider three versions of the go tasks in our model. In the simple go task (Fig. 2c,i), the network receives a constant input during the whole trial and is expected to generate the same-magnitude signal at the output after the fixation input is turned off. In the reaction time (GoRt) task, the fixation input is supported at a constant level during the trial, and when the input stimulus is applied, the network responds by the same-magnitude output while suppressing the fixation output to zero (Fig. 2d,j). In the delayed version (GoDl) of the go task, the network receives a short input and is expected to generate the same-magnitude response at the output when the fixation input vanishes (Fig. 2e,k).

### Training procedure

Training is conducted over $N_{epoch}$ epochs. In each epoch, the network performs $N_{batch}$ randomly selected target tasks in parallel from the tensor of tasks $A_{task} \in {\mathbb {R}}^{N_{step} \times N_{batch}\times N_{in}}$, where $N_{step}$ is the number of time steps and $N_{in}$ is the number of inputs. As a normal process in supervised learning methods^42^, the produced network outputs are compared at the end of each epoch to the target values, and the mean-squared error over the tensor is computed as follows:

5 $$\begin{aligned} \text {MSE} = \frac{\sum \nolimits _{i=1}^{N_{out}} \sum \nolimits _{l=1}^{N_{batch}} \sum \nolimits _{n=1}^{N_{step}} m_{il}(n) \left[ y_{il}(n) - {\hat{y}}_{il}(n)\right] ^2}{N_{out} N_{batch} N_{step} \sum \nolimits _{i=1}^{N_{out}} \sum \nolimits _{l=1}^{N_{batch}} \sum \nolimits _{n=1}^{N_{step}} m_{il}(n)}, \end{aligned}$$

where $y_{il}(n)$ is the *i*-th network output in the *l*-th batch at moment *n*, $\hat{y}_{il}(n)$ is the corresponding target output, and $m_{il}(n)$ are the entries of the mask matrix *M*. The values $m_{il}(n)$ equal 1 during the stimulus phase of the trial and 5 during the response phase.

The mean-squared error (5) (MSE) is the first modification applied to the loss function in this work. The second (complete) loss function modification is calculated as

6 $$\begin{aligned} E = MSE + \lambda E_r, \end{aligned}$$

where $\lambda E_r$ is the regularization term. In this case, the following can be defined:

$$\begin{aligned} E_r = \sum _{i=0}^{N} f_i H(f_i - f_{th}), \end{aligned}$$

where $f_i = [1/(N_{batch} N_{time})]\sum _{l=1}^{N_{batch}}\sum _{n=0}^{N_{time}} z_{il}(n)$ is the average firing rate of the *i*-th neuron, and $f_{th} = 30$ Hz is the threshold frequency. Such regularization allows us to set a top limit on the neural firing rates exceeding $f_{th}$, thus reducing the average network activity. For this type of loss function, the parameter $\tau _{out}$ is set to $50\text {ms}$, $V_{th}=0.45$ mV, $m_{il}(n) = 0.1$ for the stimulus phase and $m_{il}(n) = 1$ for the response phase. For the loss function without regularization, $m_{il}(n)$ equals 1 during the stimulus phase and 5 during the response phase, while $\tau _{out}=2\text {ms}$.

After computing the loss function, the network weights are modified according to the stochastic gradient descent algorithm, where the error is decreased in the opposite direction of the loss function gradient. To do this, the partial derivatives of the loss function are calculated with respect to the weights, and the latter are updated after each epoch as follows:

7 $$\begin{aligned} \begin{aligned} w_{kj}^{rec}(p)&= w_{kj}^{rec}(p - 1) - \eta \left( \frac{\partial E}{\partial w_{kj}^{rec}}\right) (p - 1), \\ w_{jk}^{in}(p)&= w_{jk}^{in}(p - 1) - \eta \left( \frac{\partial E}{\partial w_{jk}^{in}}\right) (p - 1), \\ w_{kj}^{out}(p)&= w_{kj}^{out}(p - 1) - \eta \left( \frac{\partial E}{\partial w_{kj}^{out}}\right) (p - 1), \\ b_j^{out}(p)&= b_j^{out} - \eta \left( \frac{\partial E}{\partial b_j^{out}}\right) (p - 1), \end{aligned} \end{aligned}$$

where *p* is the epoch iteration step and $\eta$ is the learning rate.

We further applied the so-called surrogate gradients^43^ to prevent the problem of discontinuity in the spiking dynamics by taking pseudoderivatives according to the superspike method^44^ for the points where the real derivative does not exist:

8 $$\begin{aligned} \sigma _j' = (1 + |\alpha (v_j - v_{th})|)^{-2}, \end{aligned}$$

where $\alpha =100$ is the scaling parameter that defines the sharpness of the approximate derivative. Therefore, the derivatives of the spiking variables are approximated as follows:

9 $$\begin{aligned} \begin{aligned} \frac{\partial z_j(n)}{\partial w_{kj}^{rec}}&\approx \sigma _j'(V_j(n))\frac{V_j(n)}{\partial w_{kj}^{rec}}, \\ \frac{\partial z_j(n)}{\partial w_{kj}^{in}}&\approx \sigma _j'(V_j(n))\frac{V_j(n)}{\partial w_{jk}^{in}}. \\ \end{aligned} \end{aligned}$$

The derivatives of the membrane potentials and adaptation variables are calculated as follows:

10 $$\begin{aligned} {\left\{ \begin{array}{ll} \frac{\partial V_j(n)}{\partial w^{rec}_{kj}} &{} = \frac{\partial V_j(n-1)}{\partial w^{rec}_{kj}}\left[ 1 + \frac{\Delta t}{\tau _m}\left( \exp {\left\{ \frac{V_j(n-1) - V_{th}}{\theta }\right\} } - 1\right) \right] + \frac{\Delta t}{\tau _m}(z_j(n-1) \\ &{}\quad - \frac{\partial a_j(n-1)}{\partial w^{rec}_{kj}}), \\ \frac{\partial a_j(n)}{\partial w^{rec}_{kj}} &{} = \frac{\partial a_j(n-1)}{\partial w^{rec}_{kj}}\left( 1 - \frac{\Delta t}{\tau _a}\right) + \frac{\Delta t}{\tau _a} a_{current} \frac{\partial V_j(n-1)}{\partial w^{rec}_{kj}}, \end{array}\right. } \end{aligned}$$

and

11 $$\begin{aligned} {\left\{ \begin{array}{ll} \frac{\partial V_j(n)}{\partial w^{in}_{jk}} = \frac{\partial V_j(n - 1)}{\partial w^{in}_{jk}} \left[ 1 + \frac{\Delta t}{\tau _m}\left( \exp {\left\{ \frac{V_j(n-1) - V_{th}}{\theta }\right\} } - 1\right) \right] + \frac{\Delta t}{\tau _m}\frac{i_j^{in}(n)}{\partial w_{jk}^{in}}, \\ \frac{\partial I_j^{in}(n)}{\partial w_{jk}^{in}} = u_k(n). \end{array}\right. } \end{aligned}$$

To improve the learning performance, we use the Adam training method^42,45^ with standard coefficient values and consider three learning rate values: $5\times 10^{-2}$, $5\times 10^{-3}$, and $5 \times 10^{-4}$. The best performance is achieved when the learning rate is $5\times 10^{-3}$. For training, we use the “Norse” library^46^ based on a popular “PyTorch” machine learning framework^47^. This library allows one to apply the backpropagation method for spiking neural networks by modifying the optimization parameters.

The trial parameters used for training to perform all the target tasks are shown in Table 2. For all the tasks, there is a stimulus phase with duration $T_{stim}$ and a response phase with $T_{resp}$. In general, the first phase is defined by $u_{fix}=1$, and the second phase is defined by $u_{fix}=0$. The only exception is the delayed go/no-go task, where the fixation input does not vanish and the response moment is indicated by the incoming stimulus. For the two target tasks (*Romo* and *GoDl*), the delay parameter $T_{delay}$ indicates the interstimuli interval for the Romo task and the time before the response phase for the delayed go/no-go task. For the *GoRt* task, the first column indicates the fixation time $T_{fix}$ before the input stimulus, and an output response is expected.

**Table 2:** Parameters of the target cognitive tasks.

Task	$T_{stim}$ or $T_{fix}$, ms	$T_{delay}$, ms	$T_{resp}$, ms	$a_{stim}$
DM	${\mathscr {U}}(300, 1800)$	–	250	${\mathscr {U}}(0, 1)$
CtxDM	${\mathscr {U}}(300, 1800)$	–	250	${\mathscr {U}}(0, 1)$
Go	${\mathscr {U}}(300, 1800)$	–	250	${\mathscr {U}}(\{0, \frac{1}{7}, \frac{2}{7},..., \frac{6}{7}, 1 \})$
GoRt	${\mathscr {U}}(300, 1800)$	–	1500	${\mathscr {U}}(\{0, \frac{1}{7}, \frac{2}{7},..., \frac{6}{7}, 1 \})$
GoDl	${\mathscr {U}}(200, 600)$	${\mathscr {U}}(200, 1700)$	250	${\mathscr {U}}(\{0, \frac{1}{7}, \frac{2}{7},..., \frac{6}{7}, 1 \})$
Romo	${\mathscr {U}}(200, 600)$	${\mathscr {U}}(200, 1700)$	250	${\mathscr {U}}(0, 1)$

The term ${\mathscr {U}}(a, b)$ indicates the continuous uniform distribution of a random variable from *a* to *b*, and ${\mathscr {U}}(\{a_1, a_2,\ldots , a_n\})$ denotes the discrete uniform distribution of variables $a_i$, $i=1,\ldots , n$.

Thus, our spiking neural network is characterized by several important features. First, its spiking dynamics are given by the two-dimensional dynamical system—the adaptive exponential neuron—which has a rich repertoire of activity regimes compared to the typical leaky integrate-and-fire neurons. Second, we do not impose structural constraints, e.g., Dale’s principle, but rather allow the network to evolve during training toward the most optimized state. Third, it is trained to perform multiple target tasks inspired by cognitive neuroscience experiments. Fourth, we used two types of loss functions to supervise network training: a simple mean-squared error function and a function with a regularization term that penalizes the highest firing rates.

### Analysis of the trained network

**Accuracy measurement** The trained spiking network performance in completing multiple target tasks is computed as follows. There are in total 100 testing trials of randomly selected tasks for each trained network unless otherwise stated. In each testing trial, the fixation output is evaluated to determine whether its value is larger than the threshold value ($y_{fix}=0.5$). Otherwise, the trial is assumed to have failed. If the fixation output meets the criterion, the outputs $\langle y_{1,2}\rangle$ averaged over the response phase $T_{resp}$ are calculated. For the tasks of choice (*DM*, *CtxDM*, and *Romo*), the trial is considered correct if the average $\langle y_{j}\rangle$ value corresponding to the target value ${y}_j=1$ is larger than the other value. For the repeat tasks (*Go*, *GoRt*, and *GoDl*), the trial is considered correct if the absolute difference between the corresponding output average and the target value does not exceed $\approx 0.15$.

**PCA** The multidimensional spiking activity of the trained network is analyzed by the principal component method^48^ to uncover dynamic mechanisms underlying task performance. The data matrix $U_{data} \in {\mathbb {R}}^{N\times N_{times}}$ is constructed, where each row contains one particular neuron’s dynamic variable over the sequence of trials, while each column represents the states of all the neurons at one particular moment. The analysis is performed for variables $v_j$ and $a_j$, where $j=1,\ldots ,N$, separately to correctly reflect the difference between their time scales. Using this dimensionality reduction technique, we obtain three-dimensional projections of the neural network trajectories, allowing us to identify different stages of the trials and the mechanisms of task computation.

**DPCA** To determine the role of neural activity in mapping particular aspects of cognitive tasks, we adapt the method of demixed principal components^49^ (dPCA) to analyze model data. In the traditional PCA approach, projections of phase trajectories onto the subspaces with maximum data variance can be found, and the information about inputs and outputs is not taken into account. Thus, the resulting projections are characterized by mixed selectivity as the original time series of individual neurons. The essence of the dPCA method is to find a special decomposition of the data matrix ${\textbf{X}}$ that contains a time series of instantaneous firing rates. In general, matrix ${\textbf{X}}$ has *N* rows and *T* columns, where *N* is the network size and *T* is the number of discrete time steps in the trials. The network can receive *S* different stimuli, *D* different decisions can be made at the output, and for each parameter set, *K* trials are implemented. Thus, the *i*-th row contains information about the firing rate of the *i*-th neuron for different combinations of input stimuli and output responses. Decomposition of matrix ${\textbf{X}}$ results in the following expression:

12 $$\begin{aligned} {\textbf{X}} = \sum \limits _{\phi } {\textbf{X}}_{\phi } + {\textbf{X}}_{noise}, \end{aligned}$$

where the terms ${\textbf{X}}_{\phi }$ are demixed components of the population activity, two of which depend either on stimuli (${\textbf{X}}_{s}$) or only on decisions (${\textbf{X}}_{d}$). The combined component is only dependent on time (${\textbf{X}}_{t}$), and ${\textbf{X}}_{noise}$ is a noise term.

**k-means and hierarchical clustering** The k-means method^50,51^ is used to identify neural subgroups in the trained network responding in a certain way during different tasks. For each of the $N_{task}$ target tasks, $N_{trials}$ trials are carried out, and the average firing rates $f_j = \langle z_j(n)\rangle$ are calculated for each *j*-th neuron, where $j=1,\ldots ,N$. Then, the data matrix $K\in {\mathbb {R}}^{N\times (N_{task}*N_{trials})}$ is obtained, and it contains each neuron’s average rate for different tasks under various initial conditions. After that, the matrix is normalized to the maximum value of its entries. The algorithm minimizes the total quadratic deviation of the distances between the cluster points and the cluster centers or centroids:

13 $$\begin{aligned} J(C) = \sum _{k=1}^K \sum _{i \in C_k} ||f_i - \mu _k||^2 \underset{C}{\rightarrow }\ \min , \end{aligned}$$

where *C* is the set of clusters of power *K*, and $\mu _k$ is the centroid of cluster $C_k$.

To analyze the cluster partitioning of the network, we use Ward’s method for agglomerative hierarchical clustering. In this method, the distance between two Clusters A and B is determined as follows:

14 $$\begin{aligned} R(A, B) = \sum _{i\in A\cup B}(f_i - \mu _{A\cup B})^2 - \sum _{i\in A}(f_i - \mu _A)^2 - \sum _{i\in B}(f_i - \mu _B)^2, \end{aligned}$$

where $f_i$ is the firing rate averaged over different trials for each task and $\mu _j$ is the center of the *j*-th cluster. During each step of the algorithm, two clusters are merged, finally leading to a dendrogram of the hierarchical clustering structure.

## Results

### Training in detail

In all our simulations, the spiking neural network is trained with $N_{epoch}$ epochs, and in each epoch, it is presented with $N_{batch}$ randomly selected tasks. Each task is selected by changing the task coding vector ${\textbf{u}}_{tasks}$. The produced network outputs are compared at the end of each epoch to the target values, and the loss function over the epoch is computed. We use two types of loss functions in this work. The first one is a simple mean-squared error (5) function, and the second one is the sum of the mean-squared error and the regularization term, which imposes a maximum limit on the neural firing rates. Given the loss function, the network weights are modified according to the stochastic gradient descent algorithm. For the networks without regularization, $N_{batch} = 50$; for those with the regularized loss function, $N_{batch} = 32$. Each target task from 12 possible tasks is selected with a random duration of the stimulus and response phases so that to correctly assemble the tensor $A_{task}$, an individual trial duration is aligned to the longest duration in the batch. This is done by extending the trial with zero inputs before receiving the original stimuli.

For both loss functions, we calculate how the average task performance of the trained network varies with increasing number of epochs for different values of the network size and learning rate (see Supplementary Material, Fig. 1). We find that for the case without regularization, the networks containing 400–600 neurons reach a performance of more than $95\%$ after $N_{epoch} = 3000$ epochs at a learning rate of 0.005. The regularization of the firing rates enabled us to obtain similar results for smaller neuron numbers, so in our analysis, we mainly use a network with $N=256$ neurons for the loss function with regularization and $N=600$ neurons otherwise. Better performance is obtained by considering appropriate levels of noise intensity during training (see Supplementary Material, Fig. 2).

The use of the adaptive exponential neuron (2) allows us to balance the network size and its performance by varying the adaptation time $\tau _a$. This is particularly apparent for the tasks with time delays (*Romo* and *GoDl*) compared to tasks with fast spiking dynamics of model neurons without adaptation, e.g., leaky integrate-and-fire ones, because the presence of a slow variable results in greater dynamic memory. We find that better performance can be achieved when $\tau _a = 2s$ (see Supplementary Material, Fig. 3), so this value is used in most simulations.

### Network outputs

**Figure 5:**
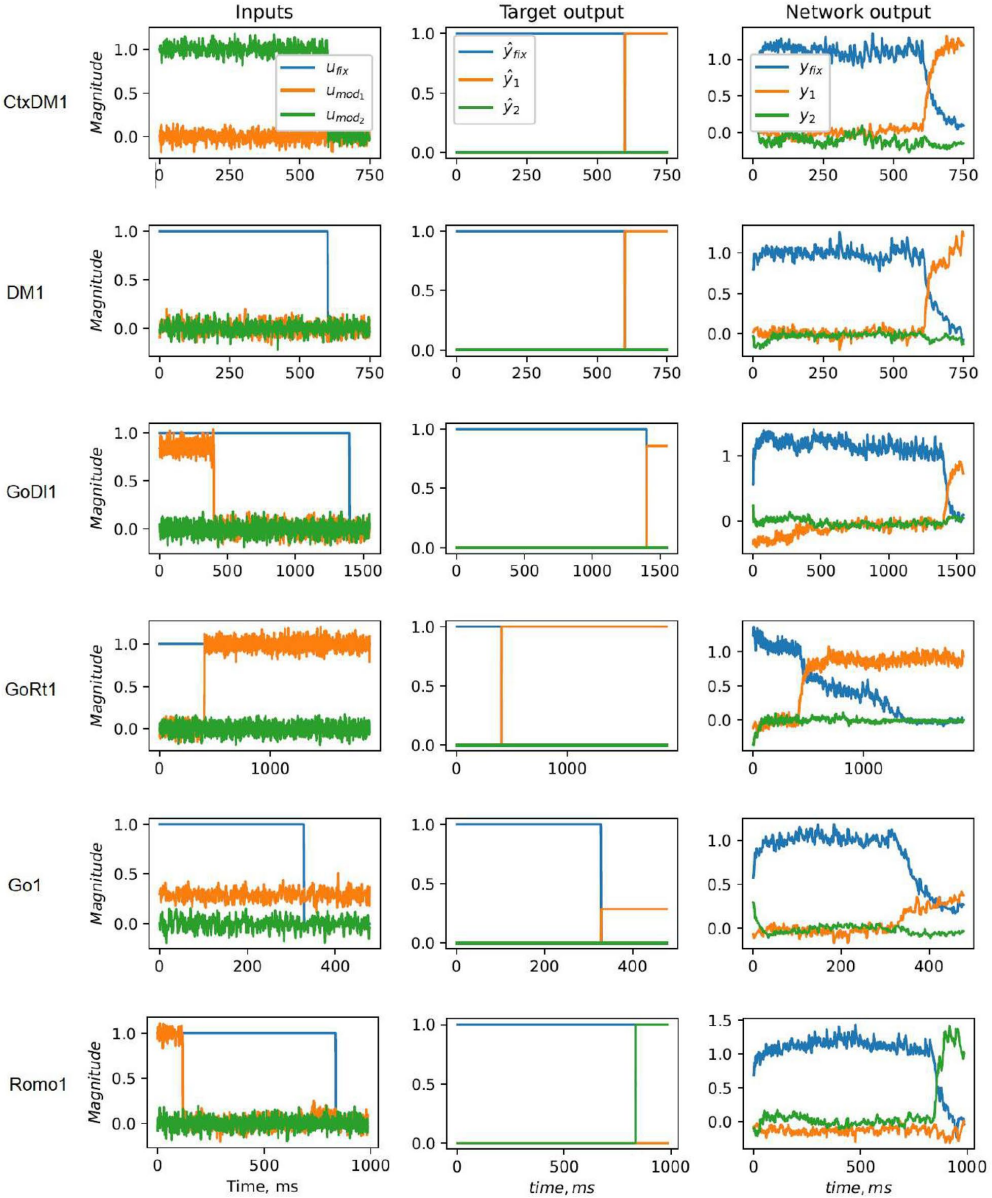
How the trained neural network performs different target tasks: the inputs (the first column) for the indicated tasks, the targets (the second column) and network output responses (the third column). The network with $N=256$ neurons is trained with the mean-squared loss function, and the other parameters are taken from Table 1.

After training, the spiking neural network is capable of completing all the target tasks with varying durations of the trial phases and different levels of input noise intensity. The performance evaluated separately for each task is the highest for the decision-making tasks and the lowest for the delayed go/no-go task. The accuracy of performing other tasks varies depending on the particular network realization. Note that the outputs $y_{1,2}$ play different roles when the network completes the choice tasks and the repeat tasks. In the former case, the target signal is binary; thus, the network is expected to respond with only one of two possible values for each output. In the latter case, the network has to memorize a continuous interval of values to properly respond. Note that with the prolongation of the response phase in the testing trials, the error may increase over time. The proper performance is observed if the response phases during testing do not exceed those used in training; see Fig. 5. Nevertheless, the trained network is robust against increasing input noise and results in filtered outputs, thus showing its generalization ability (see Supplementary Material, Fig. 4).

The two modified loss functions cause the resulting networks behave differently in the autonomous case (without inputs) and when switching between different tasks. For the case of training without regularization, the neural network exhibits chaotic spiking dynamics in the absence of inputs, thus giving rise to irregular outputs. However, when the task coding input is switched on and the sensory and fixation inputs are received, the network dynamics are regularized according to the target task, leading to very good trial performance (see Supplementary Material, Fig. 5a). Therefore, in terms of nonlinear dynamics, such recurrent spiking neural networks contain multidimensional chaotic attractors in their phase space in the absence of inputs. This endogenous random activity serves as an exploratory state when the system is expecting to receive informative inputs. The incoming stimuli switch phase-space trajectories to some metastable areas formed during training. Passing through these areas results in completing the target task. Switching between different tasks occurs by directly moving from one metastable area to another without returning to the initial chaotic regime (see Supplementary Material, Fig. 5b). Hence, after successfully completing a task, the network begins to process a new task.

In the case of neural networks trained with the regularized firing rates, zero inputs result in a silent stationary state, where the random spiking activity only emerge due to the noise in each neuron. The inputs of a target task rapidly switch the network to the metastable area responsible for that task. However, the process of outputting trajectories from this area and the process of returning to the stationary state occur much slower than the initial process because the induced spiking oscillations support the whole network activity for some time; see Supplementary Material, Fig. 5c. Switching between different tasks also occurs quite slowly, giving rise to longer transient process times when performing subsequent target tasks (see Supplementary Material, Fig. 5d).

### Task-specific neural responses

**Figure 6:**
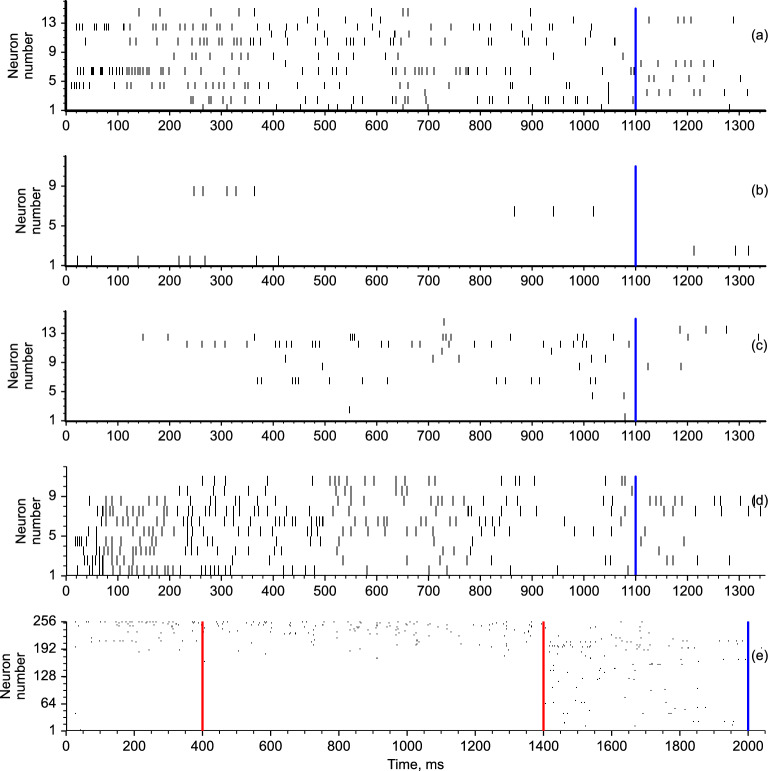
Spiking sequences of two neural subgroups of the trained network when performing the $DM_1$ task (**a**, **b**) and the $Go_2$ task (**c**, **d**). The first subgroup dynamics are given by (**a**) and (**c**), and the second subgroup dynamics are given by (**b**) and (**d**). (**e**) Full network spiking activity when performing the $Romo_1$ task.

After the spiking neural network is trained to perform multiple tasks, we analyze how individual neurons contribute to the population dynamics during the stimulus and response phases. At the single-unit level, the trained neural network exhibits spike sequences tuned to different tasks and trial phases in various ways. Neural subgroups that preferentially fire during the implementation of a particular task but are almost silent during the completion of other tasks emerge. For example, in Fig. 6a,c, the same group of neurons responds during the $DM_1$ and $Go_2$ tasks. However, in Fig. 6b,d, different neural groups respond during the same tasks. It can be seen that the neurons in the first group fire more spikes on average than those in the second group. These emergent properties are qualitatively consistent with the experimental findings in task-performing animals^16,49,52^. Figure 6e shows the full network spiking activity when completing the working memory *Romo* task. It can be seen that there are active neurons during the delay period when no input stimuli are received, and during the response phase, other neurons participate in the network output. Thus, the conclusions that the functional specialization of spiking neurons is reminiscent of the experimental observations in many neurophysiological studies of behaving animals can be made^24,49^.

**Figure 7:**
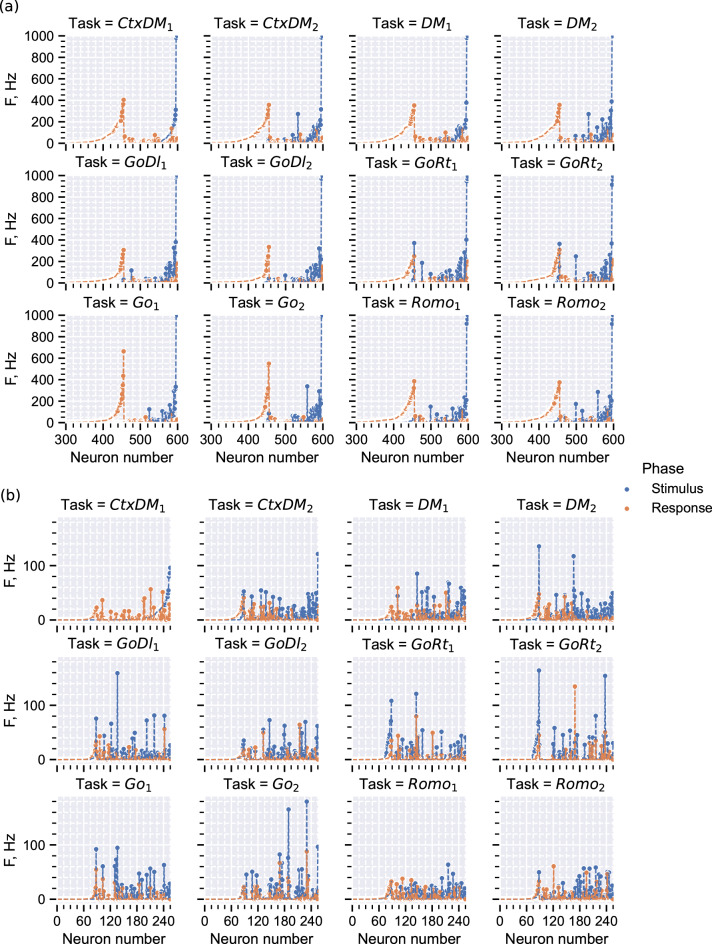
Neuronal firing rate distributions when performing different tasks during the stimulus and response phases. (**a**) Network of 600 neurons trained with the mean-squared loss function (inactive neurons not shown). (**b**) Network of 256 neurons trained with the regularized loss function. The values are obtained after averaging over 100 trials for each task and are sorted in ascending order of the rates during the stimulus phase of the first $CtxDM_1$ task.

We calculated the average firing rates of each neuron when the network performed different tasks for the two trial phases separately. There are two basic differences in the networks with the two types of loss functions. First, for the case of the pure mean-squared error, only approximately half of the neurons actively participate in the network response, and the remaining neurons remain silent. However for the regularized loss function, almost all the neurons exhibit some activity in all the tasks; cf. Fig. 7a,b. Second, the former case gives rise to very high firing rates of hundreds of Hertz of active neurons, while for the latter case, most neurons fire in the range of a few or at most dozens of Hertz. This property of spiking networks trained without regularization holds even for small numbers of neurons (see Supplementary Material, Fig. 6).

Note that for the first type of loss function, active neurons are clearly divided into sensory and motor parts, where the first part is mainly active during the stimulus phase, while the second one generates spikes during the response phase (see Fig. 7a). Moreover, this almost unchanged division is observed for all the target tasks. In contrast, for the second type of loss function, these distributions are much more complicated (Fig. 7b). In addition to the fact that this case leads to more biologically relevant firing rates and high performance at lower network sizes, the phenomenon of mixed selectivity is similar to that found in neurophysiological experiments where the neurons are not clearly tuned to one particular task or stimulus feature but rather fire under different conditions^16,49,52^.

**Figure 8:**
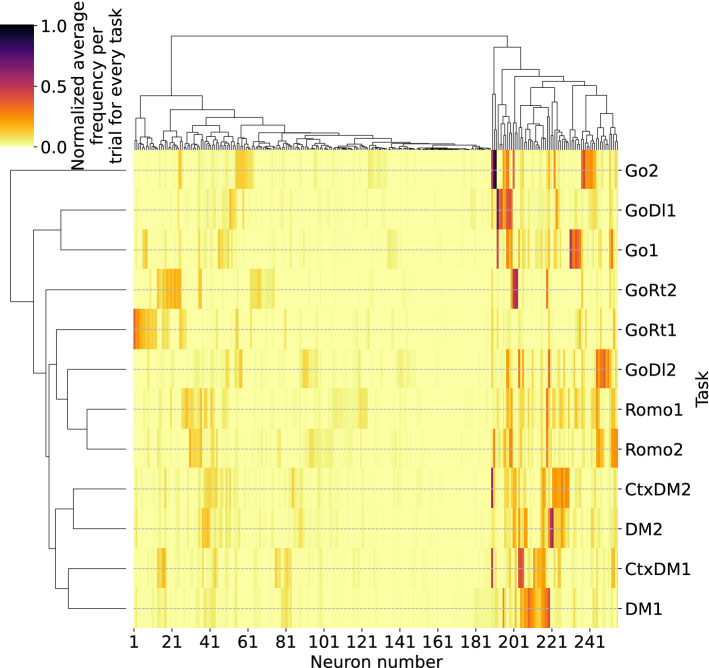
Cluster structure of the neural network after training, where the color denotes the normalized average firing rate of each neuron during each task. The dendrograms on the left and on the top show the hierarchical structures with respect to the tasks and neurons, respectively. The network consists of 256 neurons and is trained with the regularized loss function. The average of 100 test trials of each task is obtained.

To obtain more insight into how the neurons are organized after training, we applied the k-means method for detecting clusters capable of performing particular tasks and Ward’s method of hierarchical clustering. Figure 8 shows the contribution of individual neurons to different tasks, where the color intensity reflects the normalized average firing rate for different tasks and the dendrograms indicate hierarchical clustering of neurons as well as the target tasks. The dendrogram in the upper part shows two neural groups in the network, where the first largest group consists of neurons with the highest mixed selectivity that are relatively weakly active and whose activity is distributed across tasks uniformly. The second group contains clearly distinguished clusters of neurons specialized at completing particular tasks.

**Figure 9:**
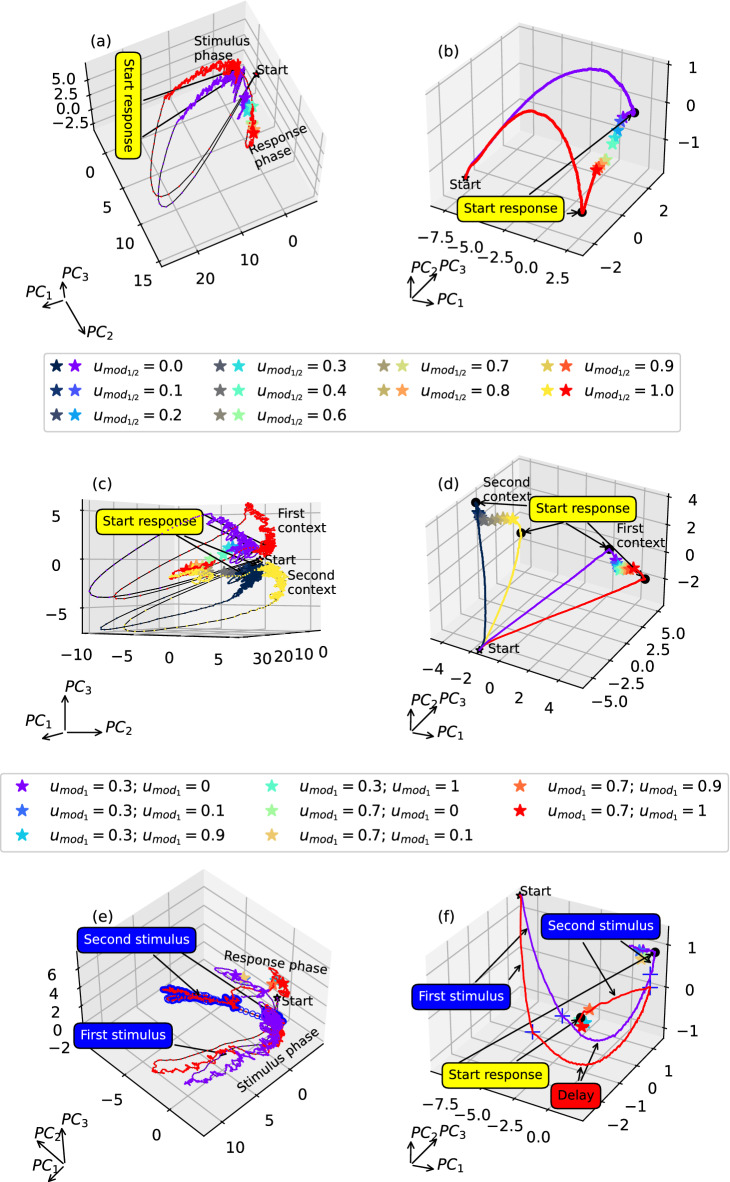
Projections of the neural activity trajectories onto the subspace of the first three principal components for variables $v_j$ (left column) and $a_j$ (right column) for the network with 256 neurons and the regularized loss function when performing the *DM* (**a**,**b**), *CtxDM* (**c**,**d**), and *Romo* (**e**,**f**) tasks. The black star indicates the beginning of each trial, and the colored stars show the termination points. The black point indicates the beginning of the response phase. Pulse symbols indicate the delay periods (**f**). Trajectories are only shown for the minimum and maximum input values for clarity. The colored asterisks in the left column show the 100th iteration of the corresponding response phase, and those in the right column show the last iteration of the response phase. The results of the network with 256 neurons trained with the regularized loss function are shown.

Therefore, the trained spiking network shows a qualitatively similar property to biological neural networks, where neurons are activated by particular stimuli or behaviors, and neurons with highly mixed selectivity exist^16,24,49,52^. The dendrogram on the left-hand side reflects the similarities and differences between the target tasks in terms of the neural activity. For example, in Fig. 8, tasks CtxDM1 and DM1 (as well as CtxDM2 and DM2) are located at neighboring branches of the dendrogram tree, showing that their implementation involves similar computational mechanisms supported by the network dynamics. Note that while the particular hierarchical structure vary in different network realizations, their basic features do not change. Similar results are observed when the network is trained without regularization (see Supplementary Material, Fig. 7). Some additional aspects of cluster identification are given in the Supplementary Material (Figs. 8, 9).

### Computation through dynamics

To study dynamic mechanisms underlying the network responses in different tasks, we analyzed projections of the high-dimensional phase-space trajectories into the low-dimensional subspace of the first three principal components. We separately considered the projections of the membrane potentials $v_j$ and the adaptation variables $a_j$, which evolved at different time scales.

In the case of the *DM* task, projections to the subspaces of both types clearly show two distinguished stages. During the first stage, the trajectories with different input stimulus values $u_{mod_1}$ slowly diverge from each other, indicating the process of sensory evidence integration, see Fig. 9a,b. During the second stage, the trajectories rapidly move to one of the two metastable states, each corresponding to the particular decision alternative. Metastability means that these are not asymptotically stable states, but they are preserved during a finite time of the response phase and disappear after that. Therefore, training induces input-specific transient trajectories in the phase space of the spiking neural network, and two sets of metastable points emerge, indicating two possible outcomes at the end of the trials. Note that this emergent dynamic mechanism is robust to substantial noise, as shown in Supplementary Material, Fig. 4.

A similar dynamic mechanism appears after training for the *CtxDM* task; however, two flows of trajectories responsible for the activation of each of the two contexts emerge (see Fig. 9c,d). Within each flow, two distinct final states corresponding to two possible decisions can be observed^53^. In all the types of decision-making tasks, a much clearer distinction of trajectories with varying input stimuli is observed for the projections onto the adaptation variable subspace. Hence, completing these tasks with high performance requires slow dynamics provided by the adaptation variable of the model neuron.

This fact is particularly supported when analyzing PCA projections for the *Romo* task shown in Fig. 9e,f. Fast spiking dynamics projected onto a three-dimensional subspace cannot form a kind of parametric memory within the delay interval (Fig. 9e). In contrast, slow-variable projections can help to explain a basic dynamic mechanism of the working memory in the spiking neural network (Fig. 9f). Varying input stimuli give rise to the divergence of trajectories in this subspace. During the delay, the trajectories slowly move and maintain their relative positions, reflecting the input differences. If the delay is extended, trajectories are attracted to a line of metastable states that support the memorized first stimulus. After receiving the second stimulus and vanishing fixation input, trajectories rapidly jump to one of two metastable attracting sets representing two corresponding decisions.

Therefore, performing all the tasks of choice requires stimulus- and context-specific transient trajectories that reflect population coding during the task implementation and two separate sets of metastable states that transiently attract the trajectories at the end of the trials. For the repeat tasks, after training, a line of metastable states representing the values from the whole range of possible inputs and corresponding outputs emerges. Qualitatively similar mechanisms are found in the network trained without regularization (see Supplementary Material, Fig. 10).

**Figure 10:**
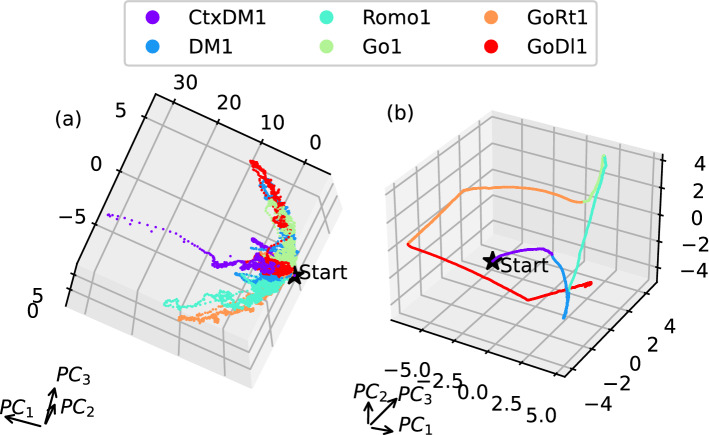
Projections of the neural activity trajectories onto the subspace of the first three principal components for variables $v_j$ (**a**) and $a_j$ (**b**) for the network with 256 neurons and the regularized loss function when performing a sequence of different tasks. The network state at the end of each trial is the initial condition for the next task.

When the network implements a sequence of alternating tasks, one observes a sequence of metastable states and transient trajectories between them in the network’s phase space, see Fig. 10. Each state corresponds to the task- and stimulus-specific output emerging at the end of the trial, and the transient trajectories reflect input the information accumulation, memory and computation resulting in a specific response.

**Figure 11:**
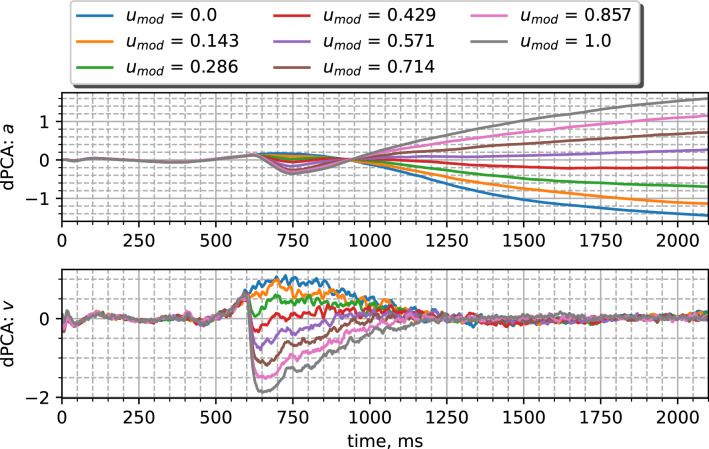
First stimulus demixed components obtained for the adaptation variables (top) and membrane potentials (bottom) during the *GoRt* task with different input stimuli. The network size is $N=256$, it is trained with the regularized loss function, and other parameters those shown in Table 1.

The method of demixed principal components allows us to obtain complementary information about dynamic mechanisms that impact task performance. Similar to the process with PCA, by applying dPCA separately to membrane potentials and adaptation variables, we determine how different phases of the trial are processed via neural dynamics. For example, Fig. 11 shows the first demixed components for the trials with increasing input stimulus when the network performs the go/no-go task with the reaction time. The early stage of the response phase (between 600 and 900 ms) is mainly determined by fast spiking dynamics given by membrane potentials, which represent the changing input. During the late stage (after 1200 ms), the memory about the input magnitude is moved into adaptation variables with slow dynamics, while the membrane potentials do not truly capture the input modification. The the initial strong network response due to the membrane potential component and the prolonged response due to the adaptation component are observed for other target tasks (see Supplementary Material, Fig. 11). Moreover, the method of demixed principal components reveals why the performance degrades for particular tasks (see Supplementary Material, Fig. 12).

### Cluster lesioning

To uncover causal properties of the neural clusters, we study the change in network performance when each cluster is lesioned and when only one cluster is active. The cluster structure is revealed, as described earlier. To lesion a certain cluster, the output weights of its neurons in matrices ${\textbf{W}}^{out}$ and ${\textbf{W}}^{rec}$ are set to zero. To make only one cluster active, only its output weights are active, and all the input links from the other clusters are set to zero.

**Figure 12:**
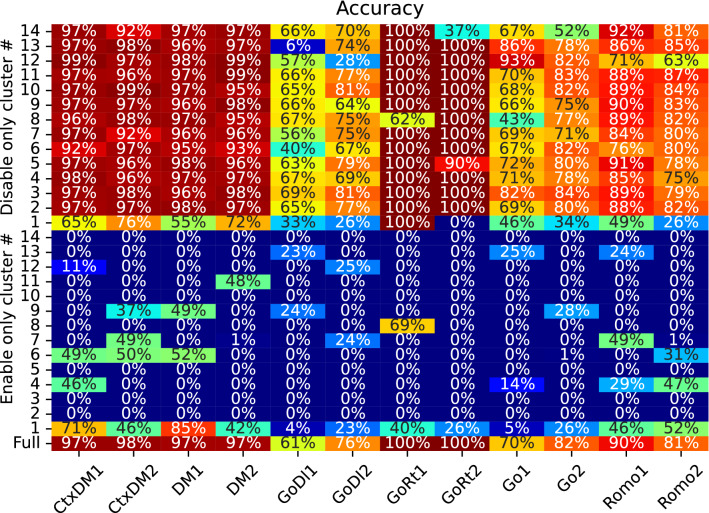
Network performance for different tasks is shown below the table when particular clusters, denoted on the left-hand side, are lesioned (top part) or switched on (bottom part). The last row indicates the mean-squared error of the original network performance for particular target tasks. Each data point is obtained for the network with 256 neurons trained with the regularized loss function after averaging over 200 test trials.

Then, the obtained networks are tested, and their performance is evaluated, as described in Sec. 2.4. The top part of Fig. 12 shows the resulting performance after particular clusters are lesioned, and the bottom part shows the performance when only particular clusters remain active in the network. The last row indicates the original network performance for the particular target tasks. Note that a cluster’s role in completing the same task for different input modalities can be quite asymmetric, reflecting the complexity of the task and stimulus representations in neuronal dynamics. For some target tasks, one or several of the most influential clusters, whose lesioning disrupts the network performance, can be distinguished, for example, Cluster 13 in the $GoDl_1$ task and Cluster 1 in $GoRt_2$. For other tasks, the importance of these terms is distributed among several or all clusters e.g., different cluster lesions cause similar performance impacts on $Go_1$.

Note that the lesion of Cluster 1 leads to the most dramatic change in performance for all the tasks except one ($GoRt_2$ in Fig. 12); thus, this cluster contains neurons with the highest mixed selectivity for the different tasks. Its isolated functioning leads to satisfactory performance compared to other clusters (see the bottom of Fig. 12). The complementary results are shown in Supplementary Material Fig. 13; the logarithmic mean-squared error function is considered and similar task performance effects are obtained. Therefore, the trained neural network is organized in such a way that the tasks can be affected by one, several or all neural clusters, and the target tasks can be regarded as one-, several- and all-cluster dependent tasks.

## Conclusions and discussion

In this paper, we presented recurrent spiking neural networks as functional models for performing multiple cognitive-like tasks. The target tasks were motivated by popular cognitive neuroscience experiments and were characterized by a stimulus-response structure. Our work extends the framework of functional neural networks, which has recently become popular in the computational neuroscience community as a promising tool for designing models with predefined constraints that, after training, are capable of completing cognitive-like target tasks. In this framework, the neural network is trained on the cognitive functions in a supervised manner, and after that, the network is reverse engineered to relate the obtained input–output mappings with features of neural activity, thus providing insight into the mechanisms of computation through dynamics.

Most of the results in this study were obtained by using rate-based neural networks for one particular target task. In our work, we designed spiking recurrent neural networks that are, on the one hand, more biologically relevant in terms of neuronal dynamics and, on the other hand, lie in the actively developing framework of neuromorphic computing, which aims at developing next-generation energy effective devices. By using supervised learning techniques, the initially random spiking networks were trained to perform multiple cognitive-like tasks depending on the task coding input. The trained neural networks were treated as multidimensional dynamical systems, and the mechanisms that impact the multitasking performance were analyzed.

Several aspects make the results of our papers distinct from previously obtained results. First, we use the adaptive exponential neuron—a more biologically relevant model than the leaky integrate-and-fire neuron. Second, the initial structure of our networks is not constrained by Dale’s principle or a modular architecture, thus allowing the network to evolve toward the most optimized state. Third, we consider a set of target cognitive-like tasks, and the network is trained to implement them all depending on the context-like signal. Fourth, two modified loss functions are used in the training procedure—the mean-squared error function without and with regularized firing rates.

We found several features that are qualitatively consistent with experimental findings observed when studying task-performing animals. First, task-specific functional clusters of neurons that fire preferentially during particular tasks or trial phases emerged. Second, some specialized spiking neurons responded to sensory inputs, while others mainly focused on producing motor outputs. Third, neurons with high mixed selectivity appeared, and they generated activity depending on various factors in a complicated manner. Fourth, we confirmed the principles of computation through dynamics, where special trajectories that emerged in the population phase space were responsible for information processing. Fifth, causal links between neural dynamics and functional properties were observed by activating and lesioning particular clusters. Note that our purpose was not to reach a quantitative resemblance between our model and any spike trains but to study if qualitative similarity appeared in the spiking network that was trained on simplified versions of cognitive tasks. Our results suggest that this is indeed the case, and our approach can serve as a springboard for future investigations.

## Supplementary Information

Supplementary Information 1.

## Data Availability

The datasets generated and analysed during the current study are available at https://github.com/Pugavkom/cgtasknet and https://github.com/Pugavkom/multy_task.
