# Supplementary material for: Multitask computation through dynamics in recurrent spiking neural networks
Source: Sci Rep. 2023 Mar 10;13:3997. doi: 10.1038/s41598-023-31110-z (PMC10006454; doi:10.1038/s41598-023-31110-z)
Supplement: Supplementary file 1 — Supplementary Information 1. [file 41598_2023_31110_MOESM1_ESM.pdf]

Supplementary Material: Cognitive multitasking through dynamics in recurrent spiking neural networks

Mechislav M. Pugavko, Oleg V. Maslennikov, and Vladimir I. Nekorkin

Institute of Applied Physics of the Russian Academy of Sciences, Nizhny Novgorod 603950, Russia

ABSTRACT

In this Supplementary Material, additional results corroborating the main content of the paper are given.

Network performance versus different parameters

Figure 1 shows how the network performance varies with increasing number of training epochs for different values of the network size, the learning rate, and the adaptation time. The maximum performance achieved for particular values of the learning rate and the adaptation time are shown to the right of the subgraphs.

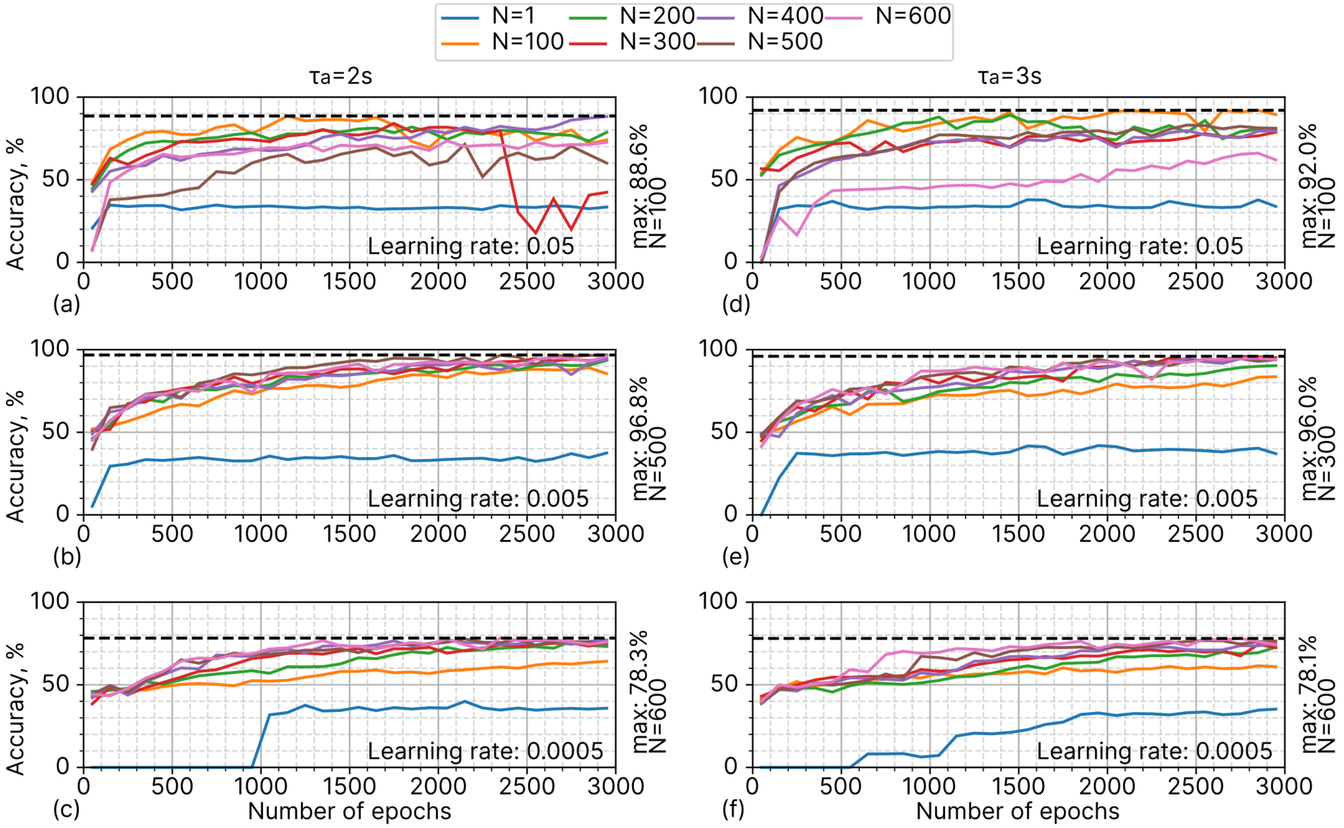

Figure 1. Network performance versus the number of training epochs for different network sizes, learning rates, and the adaptation time. The left column: $\tau_a = 2$ s, the right column: $\tau_a = 3$ s. The learning rate is equal to (a,d) $5 \cdot 10^{-2}$, (b,e) $5 \cdot 10^{-3}$, (c,f) $5 \cdot 10^{-4}$. On the right-hand side of each subplot, the maximum performance and the corresponding network size are shown.

Figure 2 shows how the network performance varies with increasing intensity of noise during testing for three particular levels of noise during training.

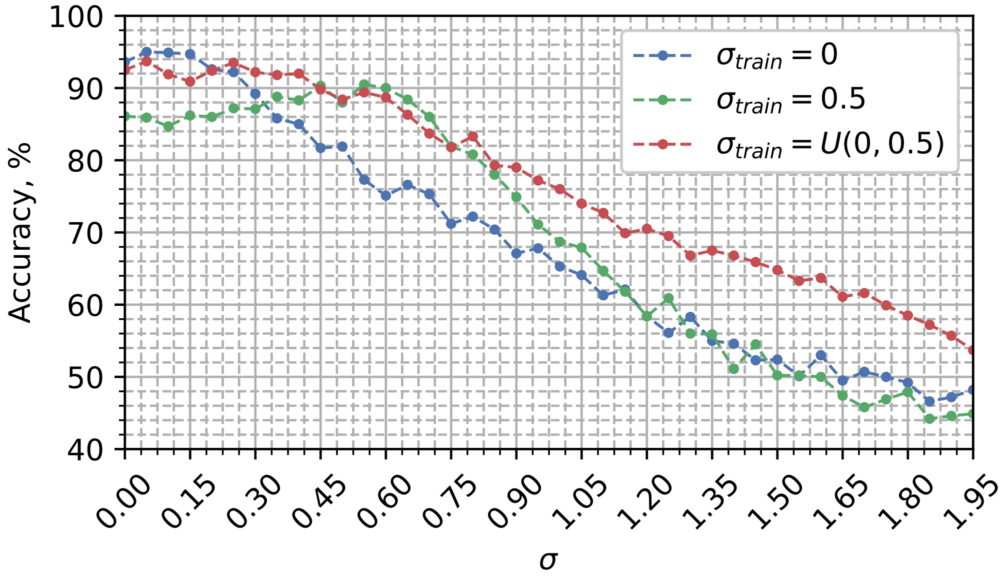

Figure 2. Network performance versus the standard deviation of the input noise σ during the testing trials for three different noise levels used during training: $\sigma_{train} = 0$, $\sigma_{train} = 0.5$, $\sigma_{train} = U(0, 0.5)$. The last one means that at each time step, the noise intensity is taken from the uniform random distribution in the interval from 0 to 0.5. The results are shown for the network of 600 neurons and each point is obtained after averaging over 500 trials.

Figure 3 shows the impact of the adaptation time τ_a on the network performance calculated separately for the tasks with and without time delay. The networks are trained with the loss function without regularization.

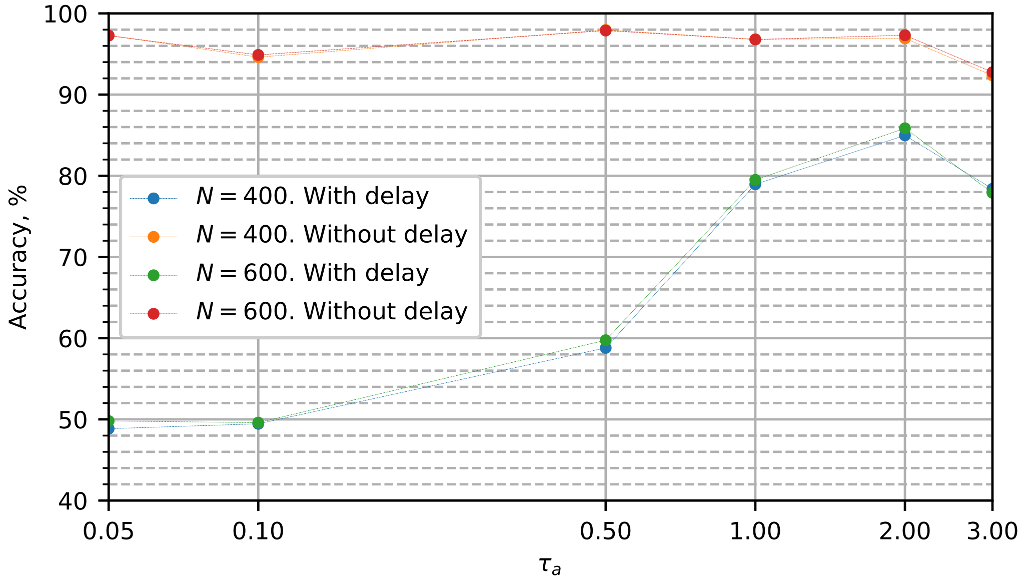

Figure 3. Network performance versus the adaptation time τ_a. The results are shown for the networks of 600 and 400 neurons and all other parameters are taken from Table 1. The performance is computed separately for the testing trials consisting of the tasks with delay (*Romo*, *GoDl*) and the tasks without delay (*CtxDM*, *DM*, *Go*, *GoRt*). Each points is obtained after averaging over 1000 trials for each task with varying input stimuli and phase durations.

Network outputs

Figure 4 shows the network outputs when performing different tasks with indicated inputs, targets, and noise intensity $\sigma = 0.6$.

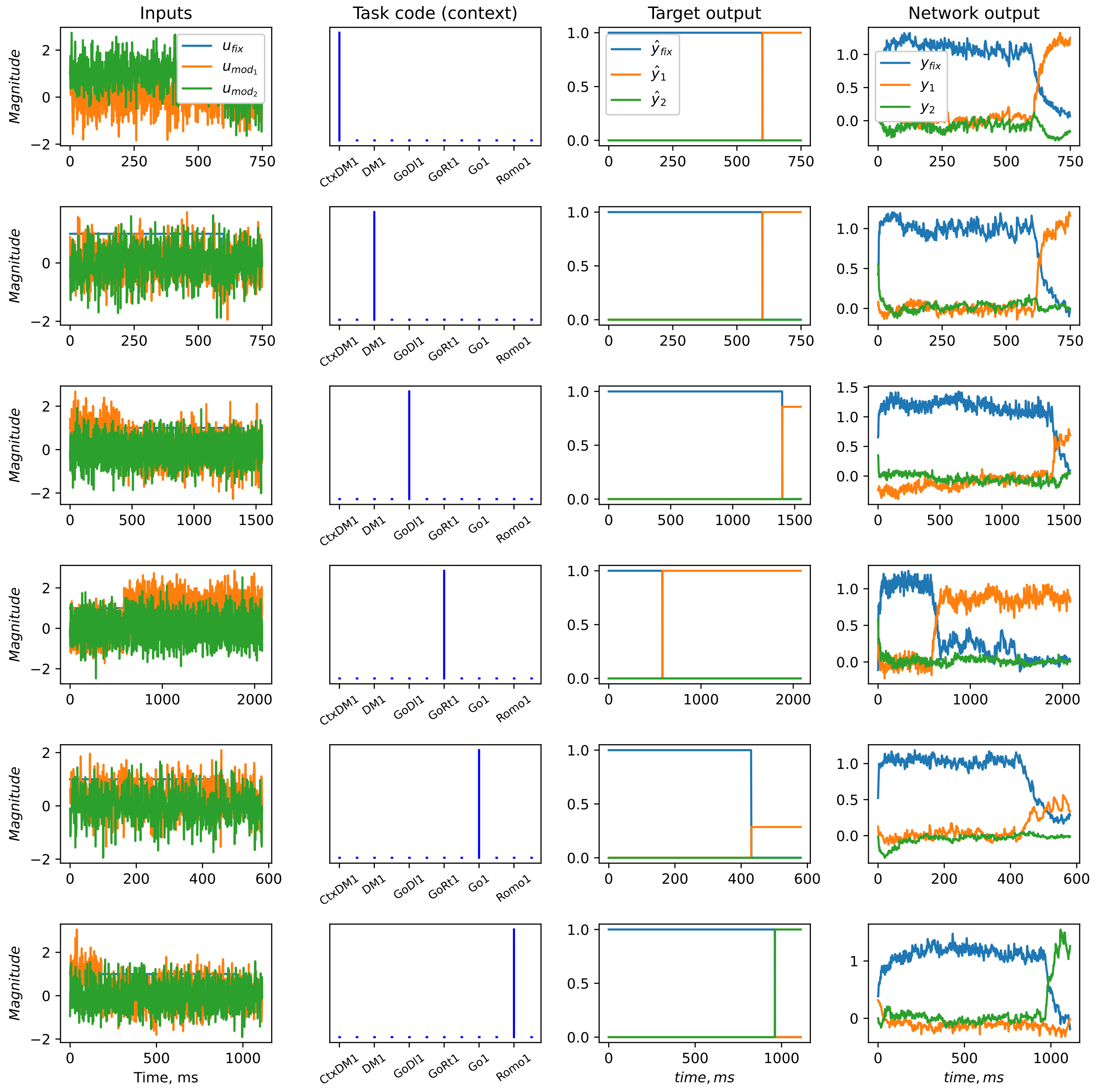

Figure 4. How the trained neural network performs different target tasks at the noise level $\sigma = 0.6$: the inputs (the first column) for the indicated tasks (the second column), the targets (the third column) and network output responses (the fourth column). Network of $N = 256$ neurons, the other parameters are taken from Table 1.

Figure 5 shows the network outputs when performing the task on the background of self-sustained activity with zero inputs and when switching between two different tasks.

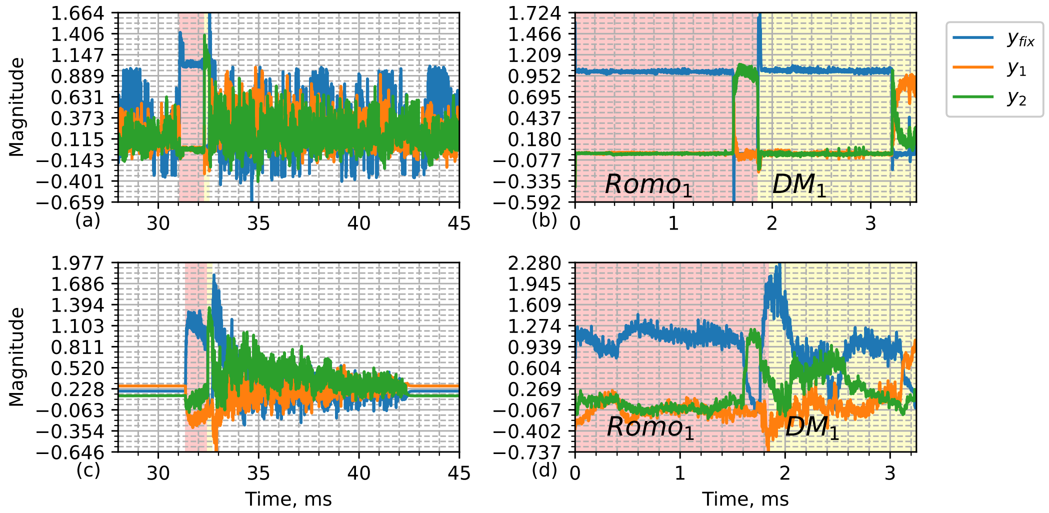

Figure 5. Outputs of the network of (a,b) 450 neurons trained without the regularization of firing rates and (c,d) 256 neurn trained with regularization. The left column shows transitions between the zero-input state of the autonomous activity to implementing *DM* task and back to the autonomous state. The right column shows switching between two different tasks *Romo* and *DM*. All other parameters was set up from table 1.

Figure 6 shows the average firing rates of each neuron when the network performs different tasks for the two trial phases separately.

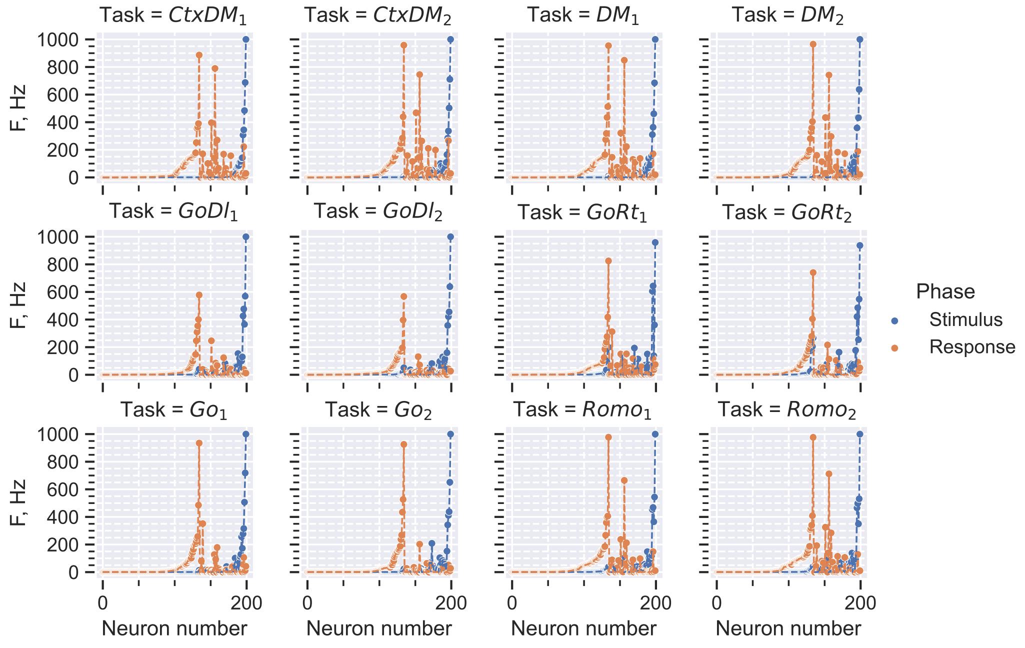

Figure 6. Neuronal firing rates distributions when performing different tasks during the stimulus and response phases. The network consists of 200 neurons and is trained with the mean-squared loss function. The values are obtained after averaging over 100 trials for each task and are sorted in ascending order of the rates during the stimulus phase of the first task.

Functional clusters

Figure 7 shows the contribution of individual neurons into different tasks for (a) the stimulus and (b) response phases where the color intensity reflects the normalized average firing rate.

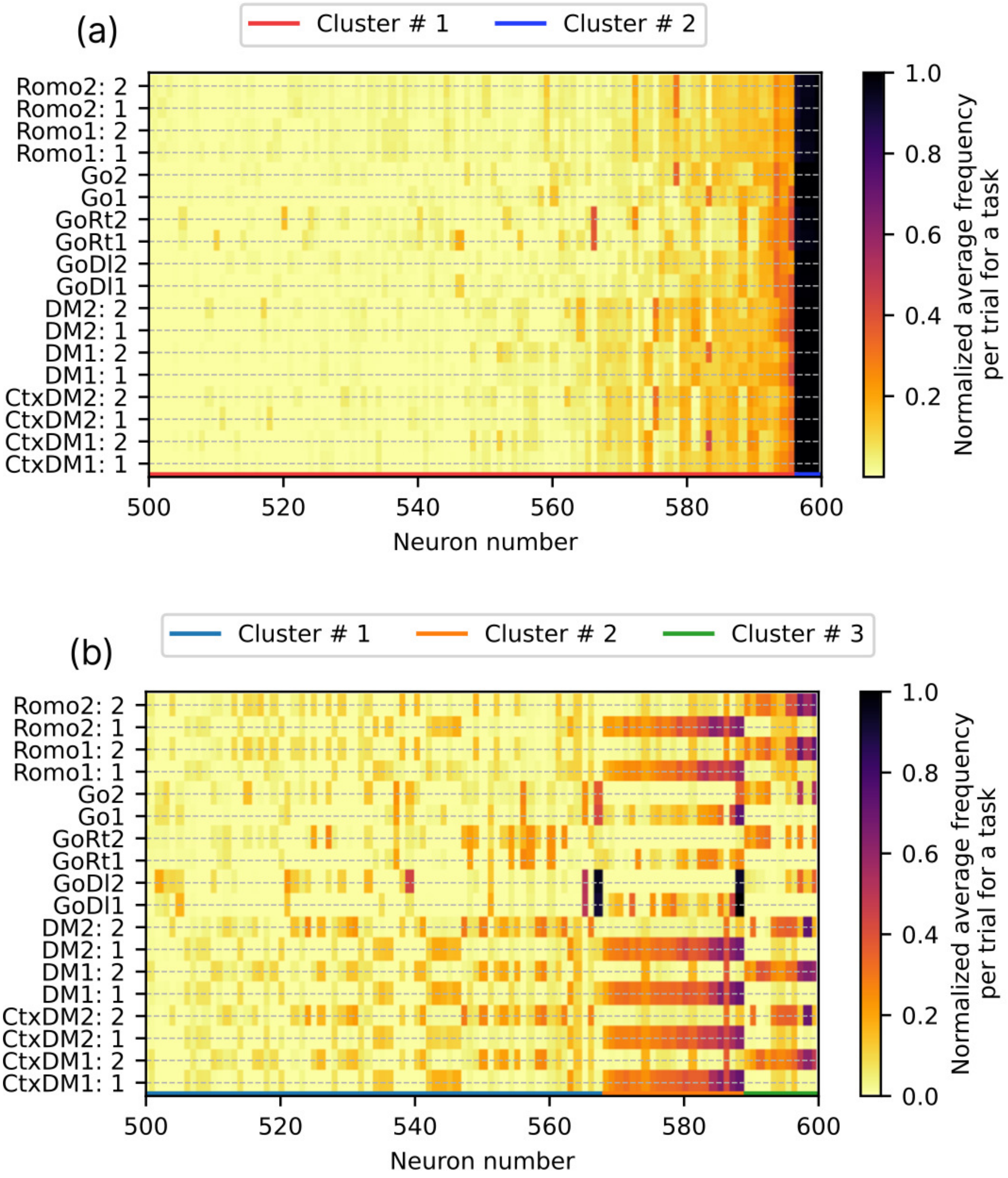

Figure 7. Functional neuronal clusters for the network of 600 neurons trained with the loss function without regularization. Top, cluster structure during the stimulus phase of the trials; bottom, during the response phase. The color codes the average firing rate of each neuron normalized to the maximum value for each task. Other parameters are taken from Table 1.

To allocate clusters with the k-means method, we plot $J(C_k)$ versus the number of clusters. Figure 8 shows the dependence of $J(C_k)$ on number of clusters for the network of 600 neurons trained without regularization and that of 256 neurons trained

with the regularization of firing rates. In the former case, this was done for the stimulus and response phases separately. It follows from Fig. 8(a), that for both phases, it is optimal to define 2-3 clusters. From analysing Fig. 8(b), we selected a total of 14 clusters.

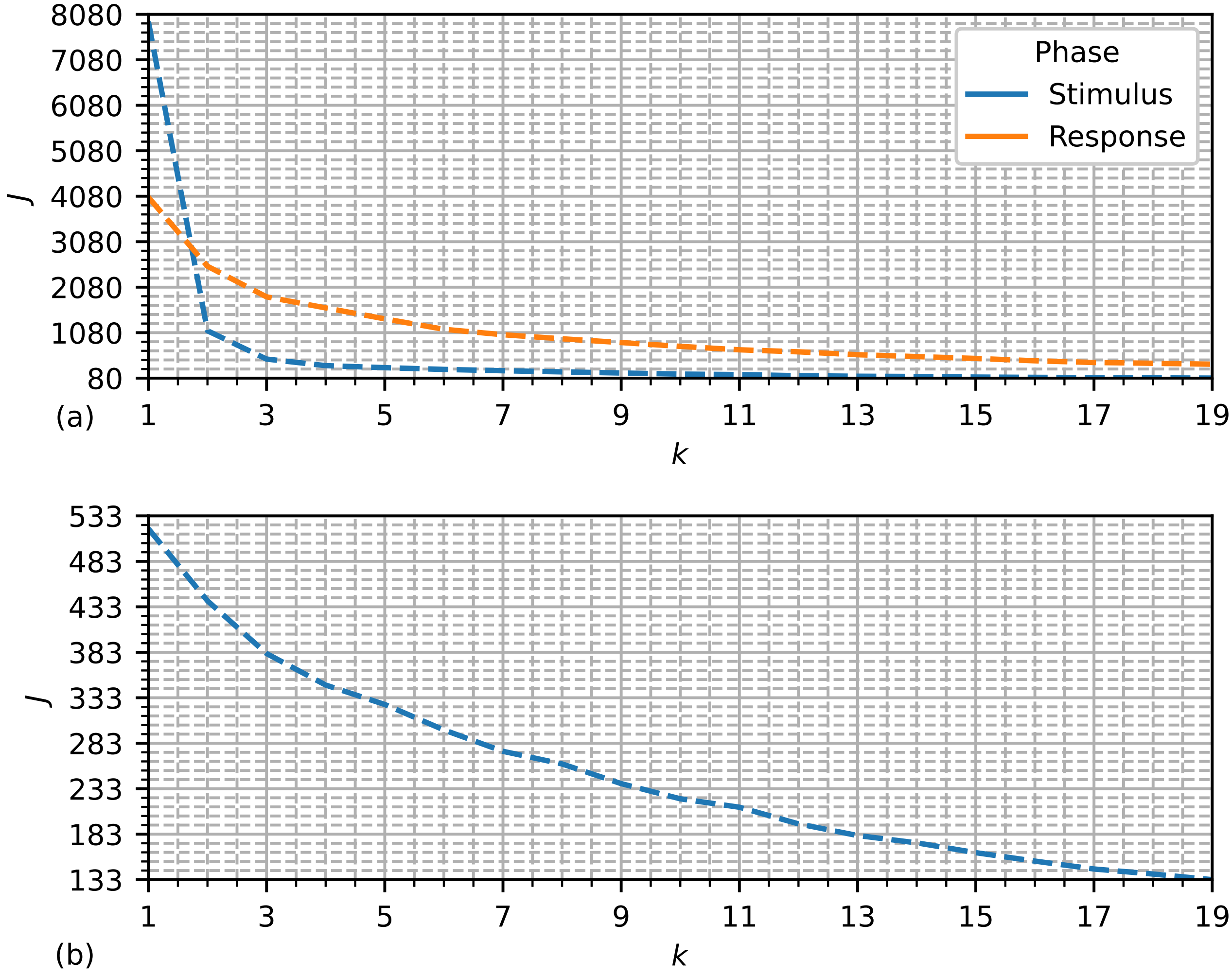

Figure 8. Quadratic deviation of the distances between the cluster points and the centroids $J(C_k)$ versus the number of clusters for (a) the network of 600 neurons trained without regularization and (b) that of 256 neurons trained with the regularization of firing rates.

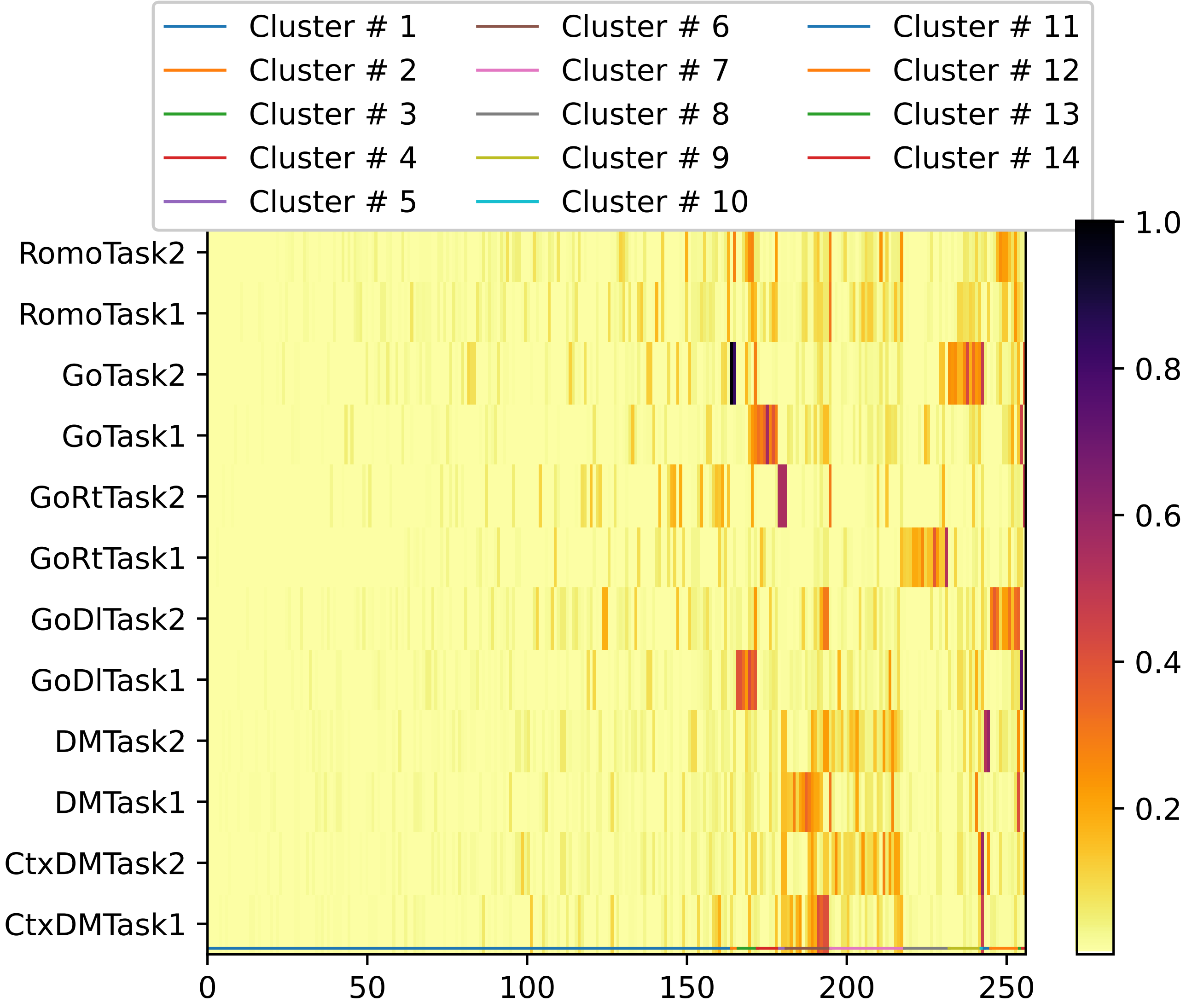

Figure 9. Cluster structure of the network of 256 neurons after regularized training where the color codes the normalized average firing rate of each neuron during each task performance. Averaging was performed over 100 test trials of each task.

Principal components

Figure 10 shows projections of the high-dimensional phase-space trajectories into the low-dimensional subspace of the first three principal components shown separately for the membrane potentials and the adaptation variables and for two different target tasks.

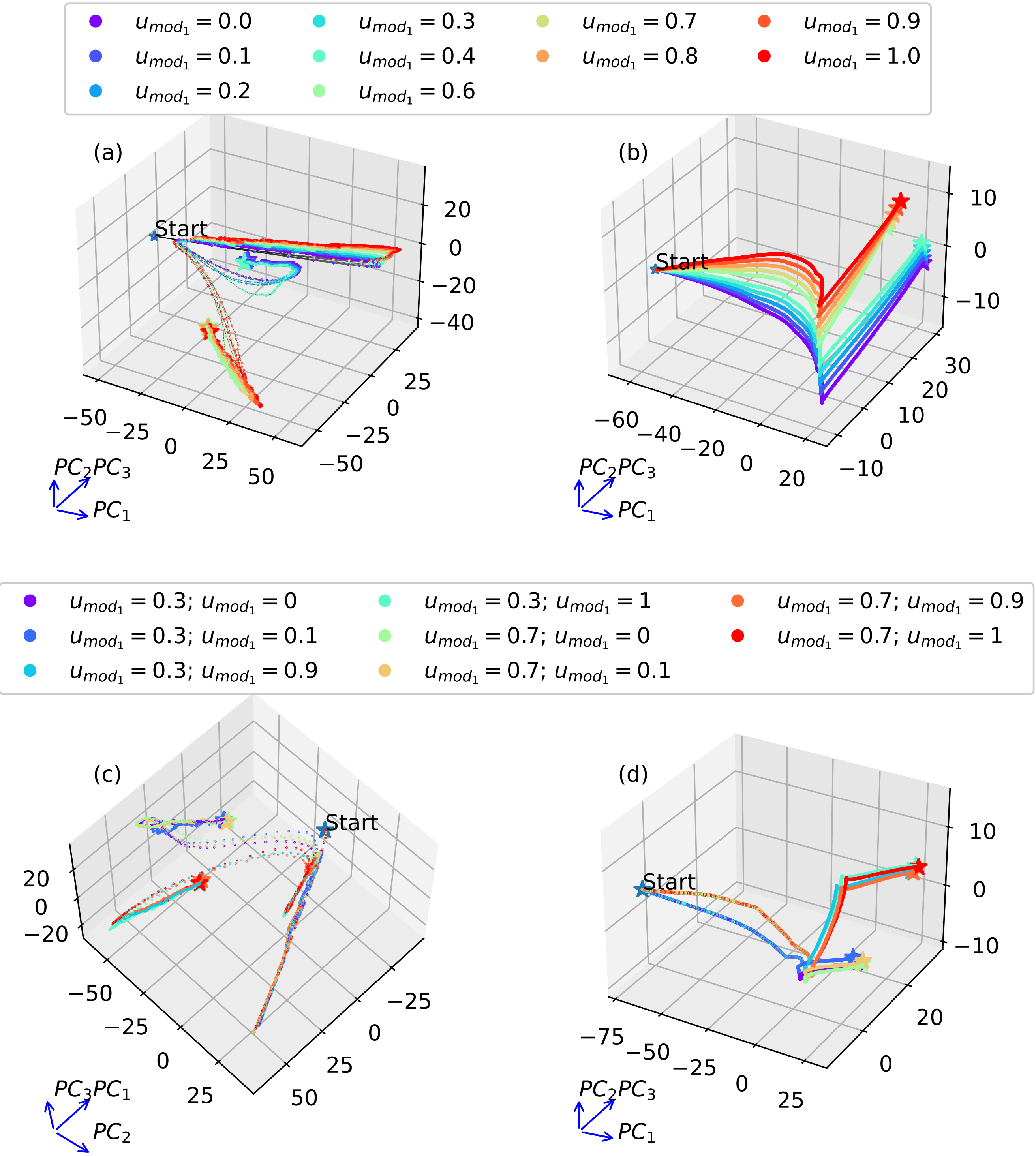

Figure 10. Projections of the neural activity trajectories into the subspace of the first three principal components for variables v_j (left column) and a_j (right column) for the network of 600 neurons trained without the regularization of firing rates when performing *DM* (a,b), *Romo* (c,d) tasks. The black star indicates the beginning of each trial and the colored stars show the termination. Other parameters are taken from Table 1.

Demixed principal components

Figure 11 shows the first demixed components for the trials with increasing input stimulus when the network of 256 neurons trained with regularization performs (a) the go/no-go task and (b) the go/no-go task with delay.

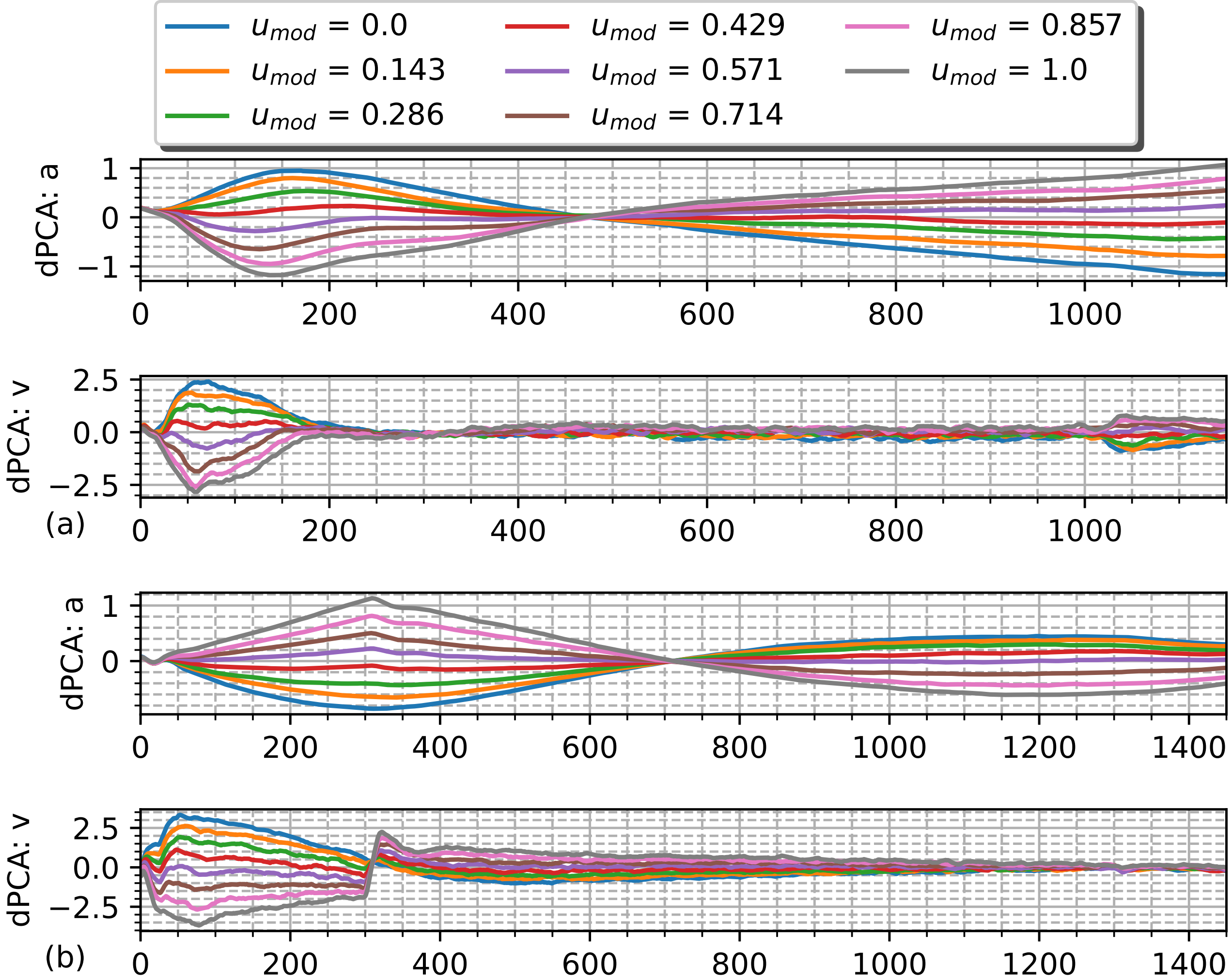

Figure 11. The first stimulus demixed components during completing (a) *Go* and (b) *GoDl* tasks for different input stimuli obtained for adaptation variables and membrane potentials. The network size is $N = 256$ and other parameters are taken as in Table 1.

Figure 12 shows the first demixed components for the trials with increasing input stimulus when the network of 600 neurons trained without regularization performs (a) the go/no-go task, (b) the go/no-go task with reaction time and (c) the working memory task.

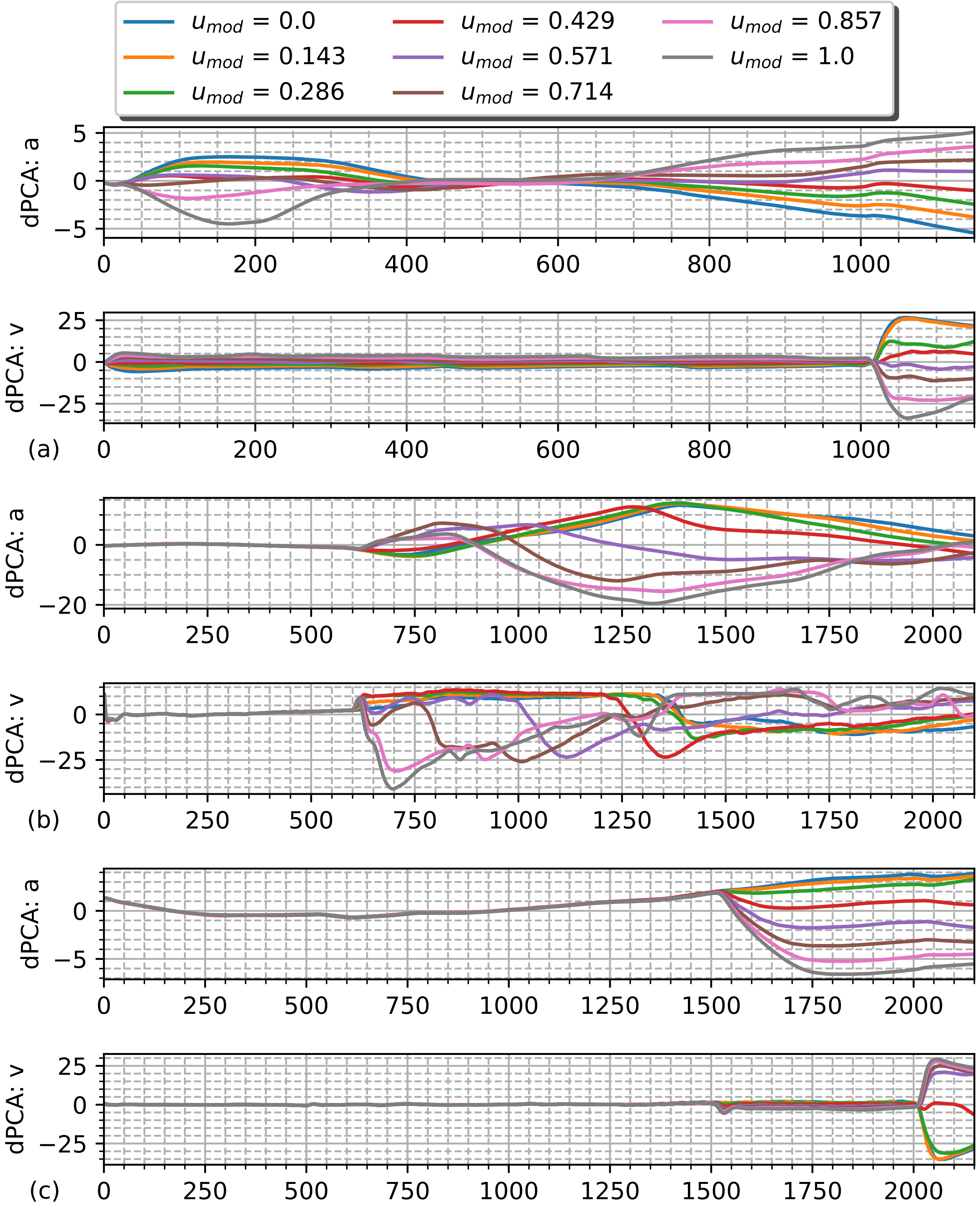

Figure 12. The first stimulus demixed components during completing (a) *Go*, (b) *GoRt*, and (c) *Romo* tasks for different input stimuli obtained for adaptation variables and membrane potentials. The network of $N = 600$ neurons is trained without regularization. Other parameters are taken as in Table 1.

Mean-squared error

Figure 13 shows the mean-squared error of the network performance after particular clusters are lesioned (top) and for the case where only particular clusters remain active in the network (bottom).

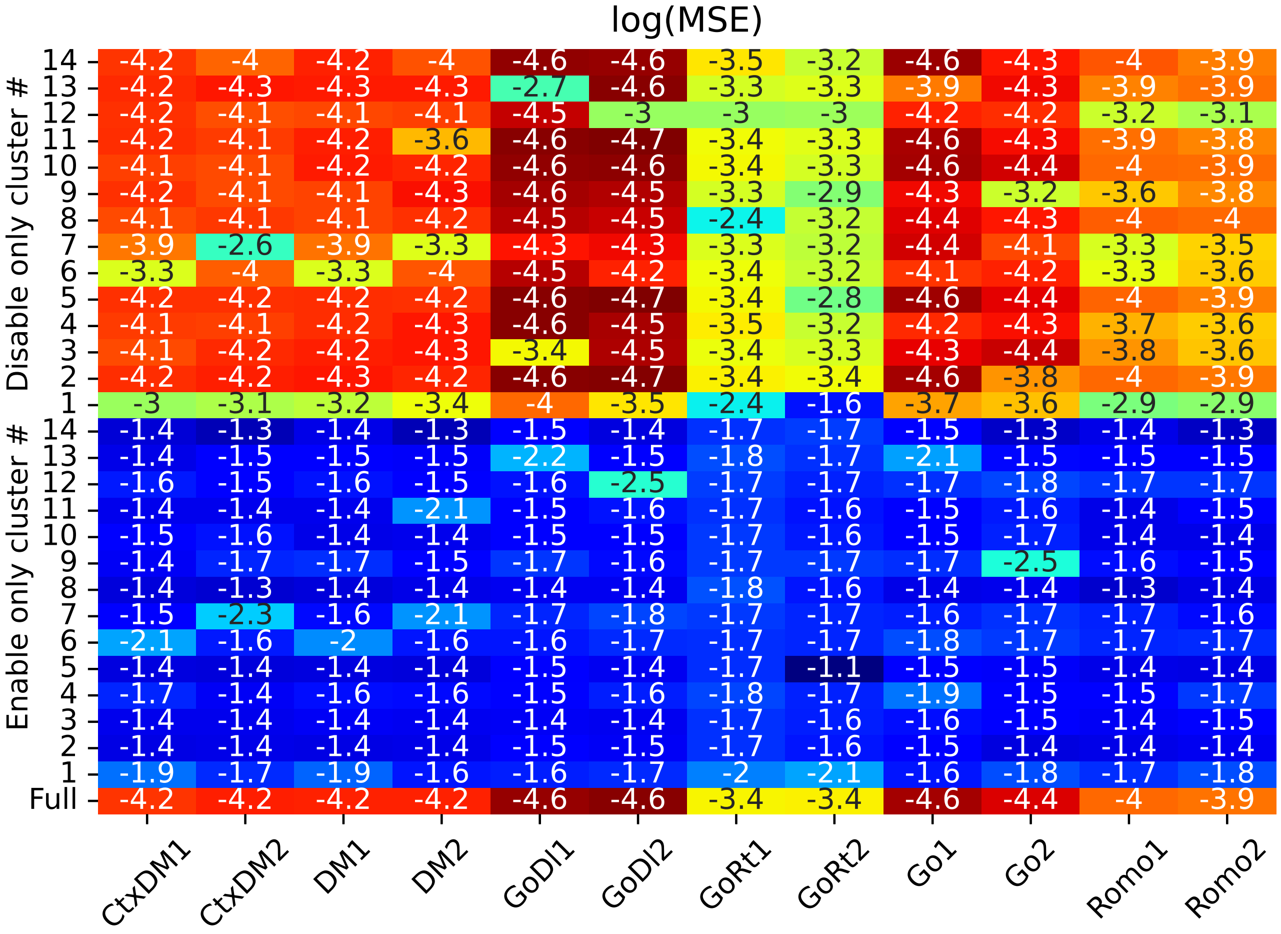

Figure 13. The mean-squared error of the network performance of different tasks shown below the table when particular clusters numerated on the left-hand side are lesioned (top part) or switched on (bottom part). The lowest row indicates the mean-squared error of the original network performance of particular target tasks.Each data point is obtained after averaging over 200 test trials.
